# GC-Rich DNA Elements Enable Replication Origin Activity in the Methylotrophic Yeast *Pichia pastoris*

**DOI:** 10.1371/journal.pgen.1004169

**Published:** 2014-03-06

**Authors:** Ivan Liachko, Rachel A. Youngblood, Kyle Tsui, Kerry L. Bubb, Christine Queitsch, M. K. Raghuraman, Corey Nislow, Bonita J. Brewer, Maitreya J. Dunham

**Affiliations:** 1 Department of Genome Sciences, University of Washington, Seattle, Washington, United States of America; 2 Department of Molecular Genetics, University of Toronto, Toronto, Canada; 3 Department of Pharmaceutical Sciences, University of Toronto, Toronto, Canada; 4 Donnelly Centre, University of Toronto, Toronto, Canada; Rutgers New Jersey Medical School, United States of America

## Abstract

The well-studied DNA replication origins of the model budding and fission yeasts are A/T-rich elements. However, unlike their yeast counterparts, both plant and metazoan origins are G/C-rich and are associated with transcription start sites. Here we show that an industrially important methylotrophic budding yeast, *Pichia pastoris*, simultaneously employs at least two types of replication origins—a G/C-rich type associated with transcription start sites and an A/T-rich type more reminiscent of typical budding and fission yeast origins. We used a suite of massively parallel sequencing tools to map and dissect *P. pastoris* origins comprehensively, to measure their replication dynamics, and to assay the global positioning of nucleosomes across the genome. Our results suggest that some functional overlap exists between promoter sequences and G/C-rich replication origins in *P. pastoris* and imply an evolutionary bifurcation of the modes of replication initiation.

## Author Summary

Genome duplication in eukaryotes initiates at loci called replication origins. Origins in most budding and fission yeasts are A/T-rich DNA sequences, while metazoan origins are G/C-rich and are often associated with promoters. Here we have globally mapped replication origins and nucleosome positions in an industrially important methylotrophic yeast, *Pichia pastoris*. We show that *P. pastoris* has two general classes of origins—A/T-rich origins resembling those of most other yeasts, and a novel, G/C-rich class, that appear more robust and are associated with promoters. *P. pastoris* is the first known species using two kinds of origins and the first known budding yeast to use a G/C-rich origin motif. Additionally, the G/C-rich motif matches one of the motifs annotated as binding sites of the human Hsf1 transcriptional regulator suggesting that in this species there may be a link between transcriptional regulation and DNA replication initiation.

## Introduction

Eukaryotic DNA replication initiates at multiple genomic loci termed replication origins. While the initiation of DNA replication at origins is a key regulatory feature of genome replication in all organisms studied, the structural components of these *cis*-acting elements are remarkably diverse [1]. Yeast origins are generally short, intergenic, A/T-rich DNA elements. In contrast, metazoan and plant origins are large, poorly-defined zones enriched for genes and G/C-rich DNA [2]–[6]. In addition, while metazoan origin activity correlates with expression of adjacent genes [2], [3], [7], no such correlation is seen in yeast. Though much has been learned about DNA replication using the highly tractable yeast models, these differences have limited the usefulness of yeast for the study of some aspects of mammalian DNA replication.

Replication origins have been best defined in the budding yeast *Saccharomyces cerevisiae*, where origin fragments shorter than 100 bp can act as autonomously replicating sequences (ARSs) sufficient for episomal plasmid maintenance [8]. The 17 bp ARS Consensus Sequence (ACS) motif is required for the interaction with the six-subunit Origin Recognition Complex (ORC) that recruits downstream initiation factors [9]. In addition to the primary ACS, origin function requires flanking DNA elements that include transcription factor binding sites [10]–[12], nucleosome depletion regions [13], [14], and helically unstable DNA [15]. While the dynamics of chromosome replication in *S. cerevisiae* are the product of a temporal timing program acting on origins with variable initiation efficiencies, the underlying regulators of replication dynamics are incompletely understood [12], [16]–[20]. Another well-studied origin model is the fission yeast *Schizosaccharomyces pombe* where longer (500 bp to 1 kb) stretches of A/T DNA are stochastically recognized by a domain of nine AT-hooks on the N-terminus of one of the ORC subunits—Orc4 [21]–[23].

Replication origins in metazoans have not been delineated to the same extent as in yeast. Metazoan replication initiates in broad replication zones that range up to 500 kb in length. Replication timing is controlled by both stochastic and regulated forces and is highly plastic throughout developmental transitions [24]. To date no clear sequence-specific binding sites for ORC have been detected in animals (or plants) though G/C-rich elements such as unmethylated CpG islands have been suggested as potential ORC targets [25]. ORC binding close to transcription start sites (TSSs) has been reported in both insects and mammals [3], [26]. Indeed there is a clear association between origin activity and local gene expression in metazoans, and the DNA viruses that infect them, which is not seen in either of the major yeast models.

Recent studies in non-canonical yeast species have elucidated that, even in related species, a diversity of consensus motifs are implicated in origin function. All budding yeast species tested so far have short A/T-rich origins with different consensus motifs. *Kluyveromyces lactis* has a 50 bp ARS consensus motif that can be accurately used to predict origin locations [27]. Conversely, *Lachancea kluyveri* recognizes sequences similar to the *S. cerevisiae* ACS, but with a much relaxed requirement for specific sequences [28]. Interestingly, its close relative *L. waltii* requires a consensus motif that bears similarities to aspects of both the *S. cerevisiae* and the *K. lactis* ACS motifs [29]. Recent profiling of replication initiation in non-canonical fission yeasts *S. japonicus* and *S. octosporus* implicated G/C-rich elements in origin function [30].

In this study we have comprehensively profiled replication origin location, structure, and dynamics in the methylotrophic budding yeast *Pichia pastoris* (*Komagataella phaffii*) [31], [32] using a number of massively parallel sequencing techniques. In addition, we generated a genome-wide profile of nucleosome occupancy. Our findings show that this yeast, which is commonly used for industrial production of recombinant proteins [33], employs at least two distinct types of DNA sequences to initiate replication. Approximately one third of *P. pastoris* ARSs require a G/C-rich motif that closely matches one form of the binding site of the well-studied Hsf1 transcriptional regulator [34]. The remaining origins use A/T-rich sequences for initiation. Genome regions near G/C-rich origins replicate significantly earlier than regions near the other class of origins and have a unique pattern of nucleosome organization. Their organization suggests that local transcriptional regulation may be linked in some way to replication timing at these sites. Furthermore, the most common plasmid vector used in *P. pastoris* contains a member of the AT-rich class of origin, suggesting that use of plasmids bearing a G/C-rich origin will yield immediate improvements for strain engineering.

## Results

### Global mapping of *P. pastoris* ARSs

**Figure 1:**
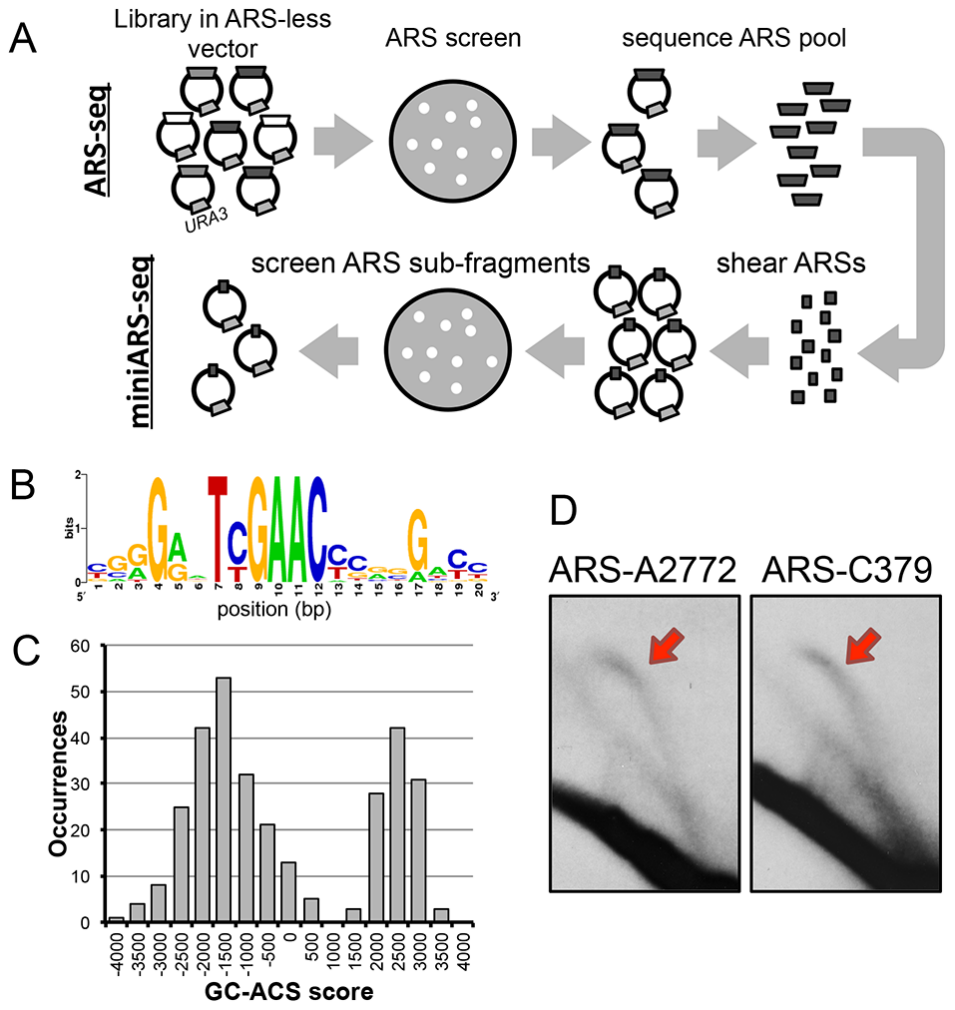
Mapping of replication origins in *P. pastoris*. (A) Schematic of ARS-seq and miniARS-seq screens. Fragmented genomic DNA was ligated into non-replicating *URA3* vectors and screened for ARS activity followed by deep sequencing of the resultant plasmid inserts (ARS-seq, top). ARS-seq plasmid inserts were amplified and sheared using DNase I. Short fragments of ARSs were ligated into the *URA3* vectors and screened for ARS activity followed by deep sequencing of the plasmid inserts (miniARS-seq, bottom). (B) The GC-ACS motif identified by the MEME algorithm. (C) The distribution of MAST motif scores of the best match to the GC-ACS in every PpARS. (D) 2D gel analysis at loci A2772 (putative AT-ARS at chromosome 1: 2,772 kb) and C379 (putative GC-ARS at chromosome 3: 379 kb). The red arrows highlight arcs corresponding to replication bubble intermediates.

The classic ARS screen identifies sequences sufficient for the initiation of replication of plasmids [35], [36] by assaying for colony formation on selective medium. Non-replicating plasmids do not yield colonies. An early study identified two regions of the *P. pastoris* genome that have ARS function, but do not have ACS elements seen in *S. cerevisiae* ARSs [37]. To generate a comprehensive map of ARSs in the genome of *P. pastoris* (PpARSs) we utilized ARS-seq, a high-throughput ARS screen combined with deep sequencing (Figure 1A) [38]. A ∼15× library of genomic DNA fragmented by one of four “four-cutter” restriction enzymes was constructed in a non-replicating *URA3* shuttle vector. A *P. pastoris ura3* strain (JC308) was transformed with this library and plated on medium lacking uracil (C-Ura) resulting in ∼20,000 colonies from an estimated 2–3×10^6^ transformants. Colonies were replica-plated on C-Ura plates and grown for four additional days before the growing colonies were pooled. Total DNA was extracted from pooled cells. ARS inserts were amplified using vector-specific Illumina primers and sequenced using paired-end deep sequencing. The sequencing reads were assembled into 971 unique genomic fragments (averaging 661 bp in length, Figure S1) and 358 overlapping contigs (Table S1). The data were filtered both computationally and by manual verification (Methods) resulting in a final list of 311 ARS loci.

To delineate the functional regions of *P. pastoris* ARSs with greater precision we used miniARS-seq, a follow-up ARS screen where the input library is constructed from short subfragments of ARSs isolated from the initial ARS-seq screen (Figure 1A) [38]. The miniARS-seq screen returned 14,661 functional ARS fragments that were filtered and assembled into contigs (Methods). This procedure narrowed the functional regions of 100 ARSseq contigs to ∼150 bp (Table S2). We have previously shown that ARS regions can be accurately narrowed by inferring functional “cores” based on regions of overlap among multiple ARS-seq/miniARS-seq fragments [38]. We combined data from both screens to generate a high-resolution map of ARS sites in the *P. pastoris* genome (Table S3).

### At least two classes of ARSs in *P. pastoris*

Identification of conserved motifs within a set of sequences with a shared function is one of the cornerstones of comparative genomics. The *S. cerevisiae* ACS motif is present in all *S. cerevisiae* ARSs and is easily recognizable by motif discovery algorithms [39]–[42]. The same is also true for *L. waltii* [29], and in *K. lactis* the ACS motif can additionally be used to predict accurately genomic ARS locations [27], [43]. We used the *de novo* motif discovery tool MEME [44] to identify conserved motifs of varying lengths within the entire set of *P. pastoris* ARSs using the zero or one occurrence per sequence (zoops) setting. MEME identified a 20 bp G/C-rich consensus motif (“GC-ACS,” E-value = 1.3e-248) with a TYGAAC core (Figure 1B). However, not all PpARSs have a significant match to this motif. To determine the subset with a GC-ACS, we used the MAST algorithm to assign a score to the best occurrence of the motif within each sequence. The bimodal distribution of motif scores (Figure 1C) indicated that 107/311 (34.4%) of the ARSs have much stronger matches to the motif than the remaining 204 ARSs. We were unable to detect any conserved motifs that were present among these 204 sequences.

We found that *P. pastoris* ARSs were significantly enriched for G/C-content relative to combined intergenic sequences (binomial P = 1.778e-06). Furthermore, the 107 ARSs bearing the GC-ACS motif (“GC-ARSs”) were significantly enriched (binomial exact test P = 2.825e-15) for G/C-content relative to the 204 ARSs without the motif (“AT-ARSs”). In fact, the AT-ARSs alone are not significantly enriched for G/C or A/T content relative to all of intergenic DNA (two-sided binomial exact test P = 0.46), suggesting that GC-ARSs are chiefly responsible for the overall G/C enrichment in the ARS dataset. Additionally, while both classes of ARSs are predominantly intergenic, GC-ARSs associate with longer intergenes whereas AT-ARSs do not. The median length of all intergenes in the *P. pastoris* GS115 strain background is 216 bp [31], whereas the median length of GC-ARS intergenes is 869 bp, an enrichment that cannot be explained by the length of intergenes alone (Monte Carlo simulation P<0.01). In contrast, the median AT-ARS intergene at 566 bp is not significantly longer than the background (Monte Carlo simulation P = 0.85). Another difference between the GC- and AT-ARSs is that the average combined ARS-seq read depths for individual ARSs of the AT- class are lower than for those of the GC-ARS class (Figure S1B, one-tailed T-test P = 0.035). This difference is most noticeable in that 61/204 AT-ARSs have a read depth <20, while all GC-ARSs have higher read depths, and only 9/107 GC-ARSs have read depths of <300. We validated a number of these low read depth AT-ARSs to ensure that they are not all false positives (Table S3). This discrepancy in read depth between GC- and AT-ARSs suggests that the AT-ARS dataset may be enriched for ARSs that replicate less efficiently in this plasmid vector context.

Similarly to other budding yeast ARSs, PpARSs are predominantly intergenic (hypergeometric test P<2.2e-16). However, unlike *S. cerevisiae*, where replication origins are enriched in convergently transcribed intergenes (where both adjacent genes are transcribed toward the intergene), *P. pastoris* ARSs are depleted in convergent intergenes (Chi-squared P = 4.749e-05, Figure S2).

To confirm that both GC-ARSs and AT-ARSs are *bona fide* replication origins in their chromosomal context, we assayed genomic origin firing by 2D-gel electrophoresis at two genomic loci (Figure 1D). Replication intermediates were isolated from exponentially growing cells in YPD medium, subjected to 2D-gel electrophoresis as described [45], and probed for a GC-ARS locus (C379) and an AT-ARS locus (A2772). The presence of an upper arc on a 2D-gel blot results from replication bubble intermediates (Figure 1D, red arrows) and is indicative of replication initiation at the probed locus. We detected such “bubble arcs” at both loci, suggesting that members of both classes of sequences can function as replication origins in the genome.

### The GC-ACS motif is required for GC-ARS function

**Figure 2:**
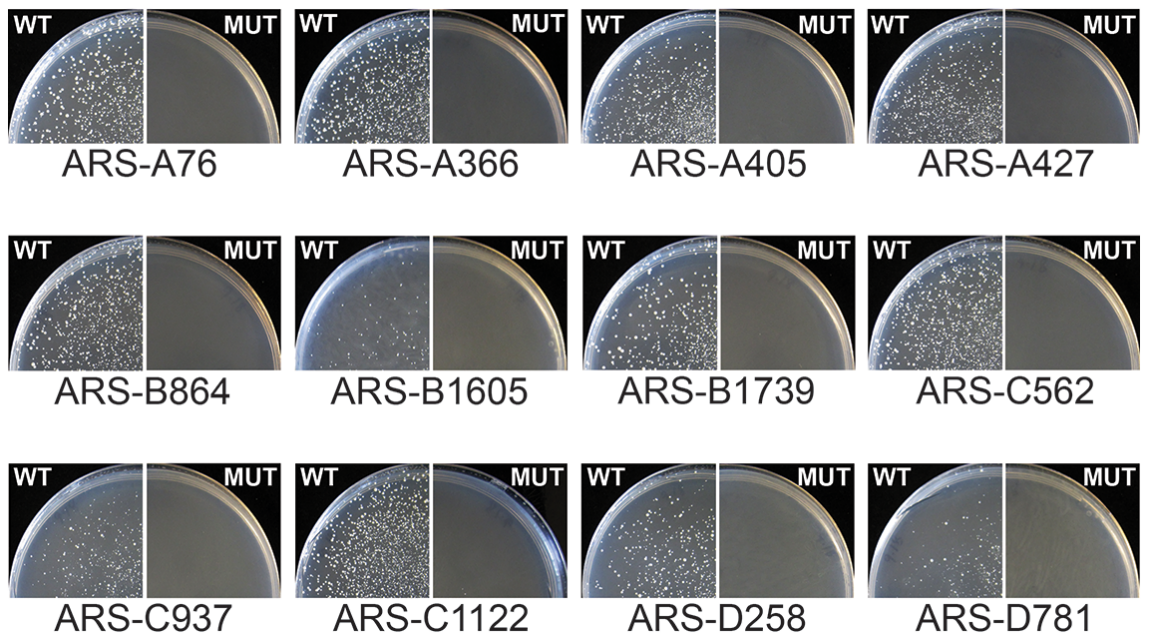
The GC-ACS is required for GC-ARS function. Wild type (WT) and mutant (MUT) alleles of the twelve ARSs indicated were cloned into a *URA3* ARS-less vector and used to transform *ura3* yeast on selective medium plates lacking uracil. Plates were grown at 30°C for five days before pictures were taken. Colony formation indicates plasmid maintenance and ARS activity. The GC-ACS was positioned <15 bp away from the 5′ endpoint in all ARS sequences. The sequences of the fragments tested are listed in Table S4.

To test whether the GC-ACS identified from the sequence analysis is required for GC-ARS function, we used site directed mutagenesis to disrupt the motif within twelve different GC-ARSs and tested the effect of these mutations on ARS function (Figure 2). We replaced the central GA dinucleotide within the best match of the GC-rich motif with a CC dinucleotide to disrupt the motif (TYGAAC was changed to TYCCAC, Table S4). We ligated short DNA fragments (125 bp) bearing both wild type and mutant alleles of each ARS into a *URA3* plasmid and tested the resulting plasmids for ARS function by transformation of the *P. pastoris ura3* strain (Figure 2). Multiple individual clones of all plasmids carrying wild-type ARS alleles yielded colonies on selective media indicating ARS activity. All clones were functional, regardless of the relative orientation of the ARS insert within the vector. Three of the twelve wild-type ARSs (ARS-B1605, ARS-C937, and ARS-D781) showed a noticeably weaker ARS activity indicated by slower colony growth. This slow growth is likely due to the short fragment length of ARSs tested, since multiple flanking elements are commonly required to support or enhance ARS function. None of the clones bearing mutant ARS alleles showed colony formation indicating the absence of ARS function independent of insert orientation within the vector. Additionally, in all twelve cases, the wildtype ARSs retained function despite the GC-ACS being positioned <15 bp from the 5′ end of the ARS fragment. These results indicate that the GC-ACS motif is required for GC-ARS function whereas sequences flanking the motif on the 5′ side are not.

### At least two distinct motifs can drive ARS function in *P. pastoris*

While the GC-ACS motif is not present in all PpARSs, the fact that it is present in over a third of ARS fragments and is essential for ARS function in the subset of GC-ARSs tested suggest that it plays an important role in ARS function. This hypothesis is further supported by the fact that ARS-seq identified most of the intergenic matches of this motif (106/134) across the genome. The remaining twenty-eight intergenic occurrences of this motif that were not detected by ARS-seq have significantly lower match scores than the motifs within ARS fragments (T-test P = 1.49e-07) suggesting that strong matches to the GC-ACS are good indicators of ARS activity.

**Figure 3:**
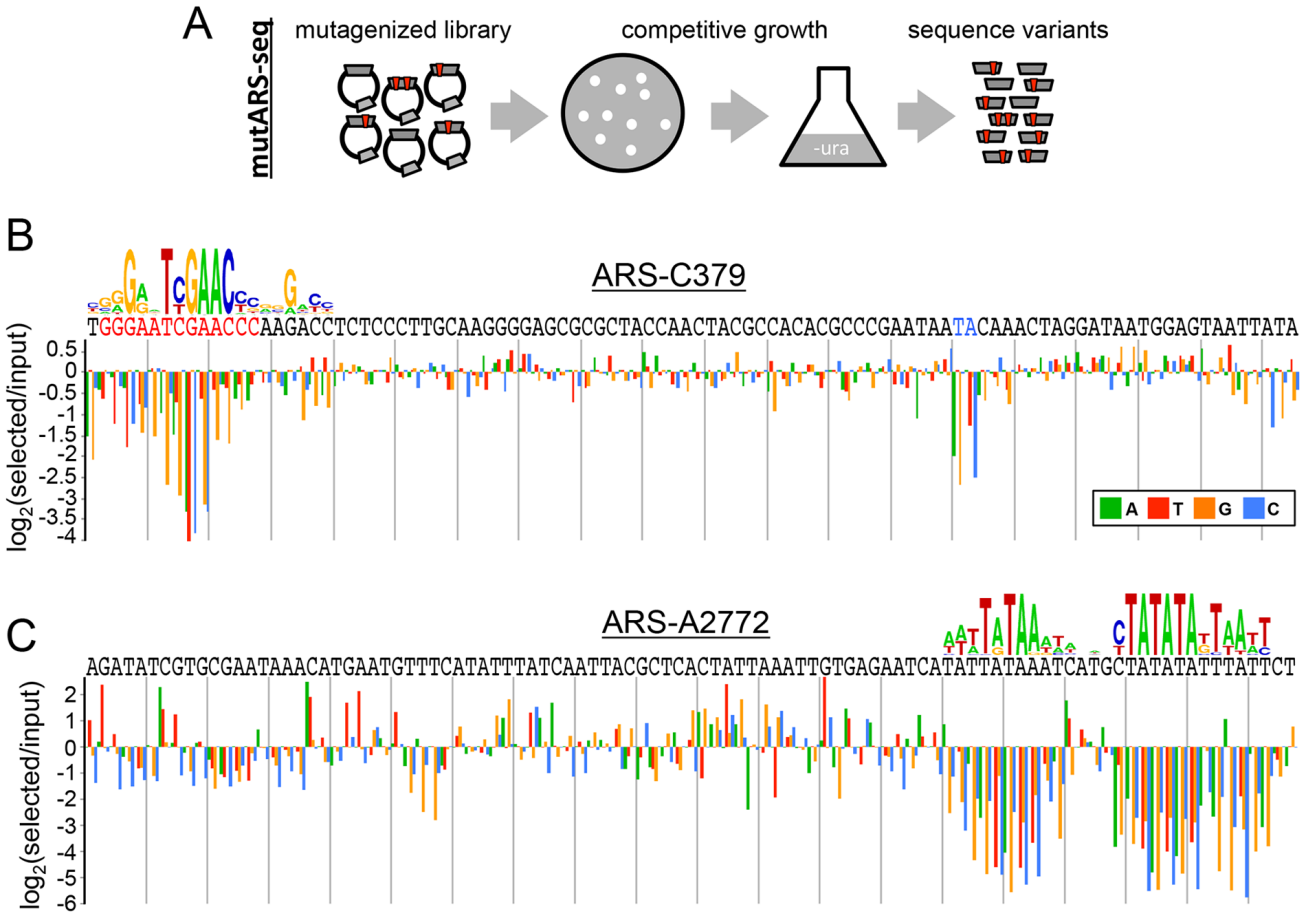
Deep mutational scanning of *P. pastoris* ARSs. (A) Schematic of the mutARS-seq deep mutational scanning experiment. Auxotrophic *ura3* yeast were transformed with a library of mutant ARS variants and competed in selective medium. The abundance of different ARS variants was determined by deep sequencing at intervals during competitive growth. (B) Results of mutARS-seq of ARS-C379. The relevant sequence of ARS-C379 is shown with the best match to the GC-ACS motif highlighted in red (and a 3′ constrained dinucleotide highlighted in blue). The log-transformed enrichment ratio is shown for each nucleotide at each position along the sequence. (C) Results of mutARS-seq of ARS-A2772. Same as in (B), except that the motif logo shown was constructed from the enrichment ratio scores post-analysis, whereas the motif shown in (B) was constructed from ARS alignments.

To assay directly the sequence determinants of ARS function, we applied a deep mutational scanning [46], [47] approach, mutARS-seq [38], to 100 bp fragments of *P. pastoris* ARS-C379 and ARS-A2772. This method involves competitively growing yeast transformed with a library of randomly mutagenized variants of a given ARS and measuring the enrichment of each allele through paired-end deep sequencing of samples over time (Figures 3A, S3, S4, and S5). Stronger ARS variants increase in population frequency over the course of the competition and are given positive enrichment scores, whereas deleterious mutations result in depletion of these alleles and are given negative enrichment scores. We constructed mutARS-seq libraries for ARS-C379 and ARS-A2772 using oligonucleotides synthesized with a 2% chance of bearing a random mutation at each position. Each library contained >20,000 inserts. A *ura3* strain of *P. pastoris* was transformed with the two libraries separately (two biological replicates for each library). Resulting colonies on selective medium plates (∼100,000 transformants for each experiment) were pooled and the cell mixture was used to inoculate a 1 L culture of liquid selective medium. The culture was grown at 30°C and the abundance of each ARS variant at different times was measured by 101 bp paired-end sequencing.

The results of mutARS-seq show a striking difference in the sequences required for function of the two types of PpARSs. ARS-C379 shows a zone of constraint within the region corresponding to the match of the GC-ACS motif (Figures 3B and S3) further supporting that the GC-ACS motif is required for ARS-C379 function. In contrast, ARS-A2772 does not have a GC-ACS and shows a region of constraint at a repetitive A/T-rich sequence that is not present in ARS-C379 (Figures 3C and S4). In searching for matches to the A/T-rich motif within the ARS set we were able to detect strong matches within only two sequences, one of them being ARS-A2772. This result suggests further complexity within the AT-ARS functional determinants. Alternatively, this motif may be inherently elusive to alignment-based methods due to its repetitive A/T-rich structure. Our findings demonstrate that *P. pastoris* can utilize at least two different non-overlapping sequence motifs for the initiation of DNA replication. We also found that these ARSs retained function in both orientations within the vector, on different length inserts, and in other plasmid contexts (data not shown), suggesting that at least one of these sequences, or an equivalent, must be present for the initiation of plasmid replication and that each is sufficient for initiation.

### GC-ARSs are earlier replicating than AT-ARSs

While the ARS assay can be used for high-precision mapping of sequences required for replication initiation, it is not an accurate measure of origin activity in the genomic context. No correlation between ARS activity and genomic replication timing has been detected in either *S. cerevisiae* or *S. pombe*, presumably due to higher-level regulation of timing that is absent on plasmids. To overcome this limitation of the ARS assay, we used an approach that combines cell sorting and deep sequencing [17], [48], [49] to map the temporal patterns of replication within the *P. pastoris* genome. This method calculates the DNA copy number ratio between S phase and G1 phase cells in sliding windows across the genome. Since a replicated region is present in twice the copy number of a non-replicated region, this copy number ratio is proportional to the relative mean replication time of a given locus [49], [50].

Approximately 1.5 million G1 and S phase cells were sorted from an exponentially growing culture using FACS. Total genomic DNA was isolated, randomly sheared, and sequenced to high coverage to measure the relative DNA copy number of all genomic loci. The ratios of sequence reads between G1 and S phase samples were calculated in non-overlapping 1 kb sliding windows across the genome and normalized based on the total number of reads within each sample (Methods). The resulting ratios from biological replicates were LOESS smoothed, yielding highly reproducible replication timing curves (Pearson and Spearman cor >0.94, Table S5). To generate a composite replication timing profile, the unsmoothed ratios from both replicates were averaged, normalized to a baseline value of 1 and smoothed (Methods, Table S5).

**Figure 4:**
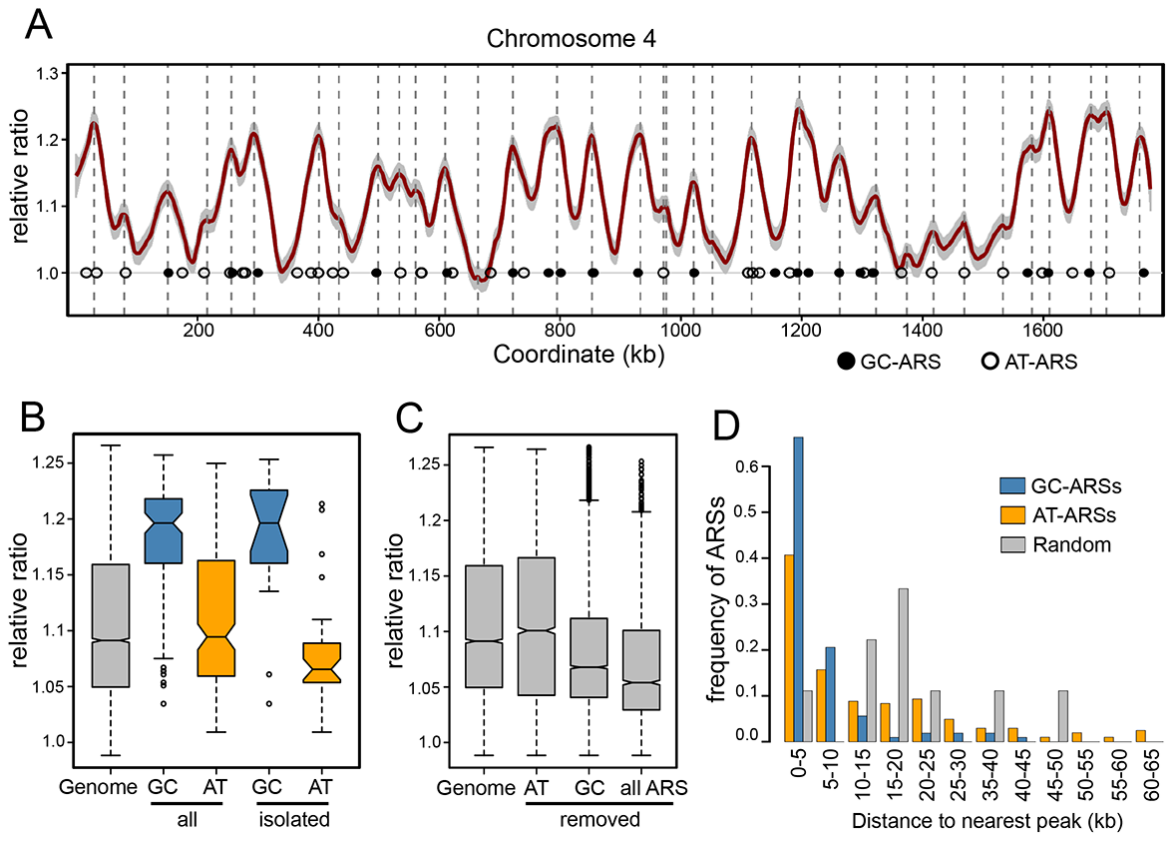
Replication timing of the *P. pastoris* genome. (A) Genomic DNA from G1 and S phase cells was sheared and sequenced. Normalized S/G1 DNA copy ratios (in 1 kbp windows) were smoothed and plotted against chromosomal coordinates. Peaks correspond to positions of replication initiation. The profile of chromosome 4 is shown (all chromosomes are shown in Figure S6) with ARS locations indicated by open (AT-ARSs) and shaded (GC-ARSs) circles. Un-smoothed ratio data for one of the replicates is shown are grey. Coordinates of replication timing peaks are indicated by dashed vertical lines. (B) The distributions of smoothed S/G1 ratio data. The distribution of all ratios (“Genome”) is shown adjacent to the distribution of values at bins containing midpoints of GC-ACSs (“GC”) or AT-ARSs (“AT”). Values for ARSs that have no other ARSs within 40 kb in both directions are shown on the right (“isolated”). (C) The complete genomic ratio distribution is shown relative to distributions after removal of data within 60 kb ranges centered on AT-ARSs (“AT”), GC-ARSs (“GC”), or all ARSs (“all ARS”). (D) For each ARS, the distance to the nearest replication peak was calculated. The ARS-peak distances are shown as distributions separately for GC-ARSs (blue) and AT-ARSs (orange). Peak distances from simulated random sets of loci are shown in grey.

Visual inspection of the chromosome replication profiles revealed ∼100 significant peaks corresponding to early replicating regions, or replication origins (Figures 4A and S6, Table S5), as well as valleys that reflect replication termination loci. Additionally, we detected numerous small peaks and “shoulders” (small peaks at the edges of larger peaks) that we interpret to be later firing or less efficient origins. Quantitative analysis identified 176 peaks in replication timing peaks (Figures 4 and S6, Table S6). Overlaying ARS coordinates with the replication curve showed that all large peaks except one contained at least one ARS. Examination of the sequence within the lone ARS-less peak (near position 1,565,000 on chromosome 1) revealed two strong matches to the GC-ACS motif within 2 kb of the peak. Manual validation of 200 bp fragments centered on each of the motif occurrences revealed them both to have ARS function indicating that they are ARS-seq false negatives. We also used the replication timing data to further validate the ARS screen to remove false positives. We manually validated low coverage ARS-seq fragments that did not appear to map at a replication peak. From forty-nine fragments with a read-depth 2–10 (fragments with read-depth 1 are filtered out at the ARS-seq stage; see Methods) eleven did not appear close to peaks and were manually tested for ARS function. Among these eleven (none of which had GC-ACS motifs), ARS activity was detected for only three (Table S4).

To test whether ARSs bearing the GC-ACS motif are regulated differently than those without, we compared the replication curve values between the two classes of ARSs (Figure 4B). Our data show that while GC-ARS regions are replicated significantly earlier than the background genomic distribution, AT-ARSs are not (T-test P<2.2e-16 and 0.0699 respectively). Consistently, GC-ARSs are replicated earlier than AT-ARSs (T-test P<2.2e-16). This result holds true even if only loci without neighboring ARSs (within a two-sided 40 kb window) are compared (T-test P = 6.267e-07). Chromosomal regions with single isolated AT-ARSs replicate significantly later relative to the pool of all AT-ARSs (T-test P = 0.0003), suggesting that clustering of these elements increases their local replication signal. This effect was not seen at the GC-ARS loci (T-test P = 0.88), indicating that clustering does not significantly affect their timing.

Another way to detect differences in replication timing between the two classes of ARSs is to measure the effect of removing their signals from the genomic dataset (Figure 4C). Removing all points within 30 kb windows centered on GC-ARSs significantly shifted the distribution of remaining replication timing signals in the “later” direction (T-test P<2.2e-16). On the other hand, removing signals around AT-ARSs did not significantly affect the distribution of remaining points (T-test P = 0.07094). When signal was removed around all ARSs, it shifted the distribution relative to removing just GC-ARSs (T-test P<2.2e-16), consistent with the AT-ARSs occupying a lower tier in the hierarchy of origin activation times.

Additionally, we found the distance from each ARS to the nearest replication peak and plotted histograms of these distances for AT- and GC-ARS's (Figure 4D). We find that both types of ARSs are significantly associated with peaks (Kolmogorov-Smirnoff test, P = 7.18×10e-5 for GC-ARSs and P = 0.0293 for AT-ARSs). GC-ARS's were significantly closer to peaks than AT-ARS's (Kolmogorov-Smirnoff test, P = 6.13×10e-7). Taken together, our data suggests that while both types of ARSs correlate with genomic replication origins, GC-ARSs are more often found associated with early origins and early replicating regions, whereas AT-ARSs show the opposite tendency.

### Nucleosome positioning at *P. pastoris* origins

**Figure 5:**
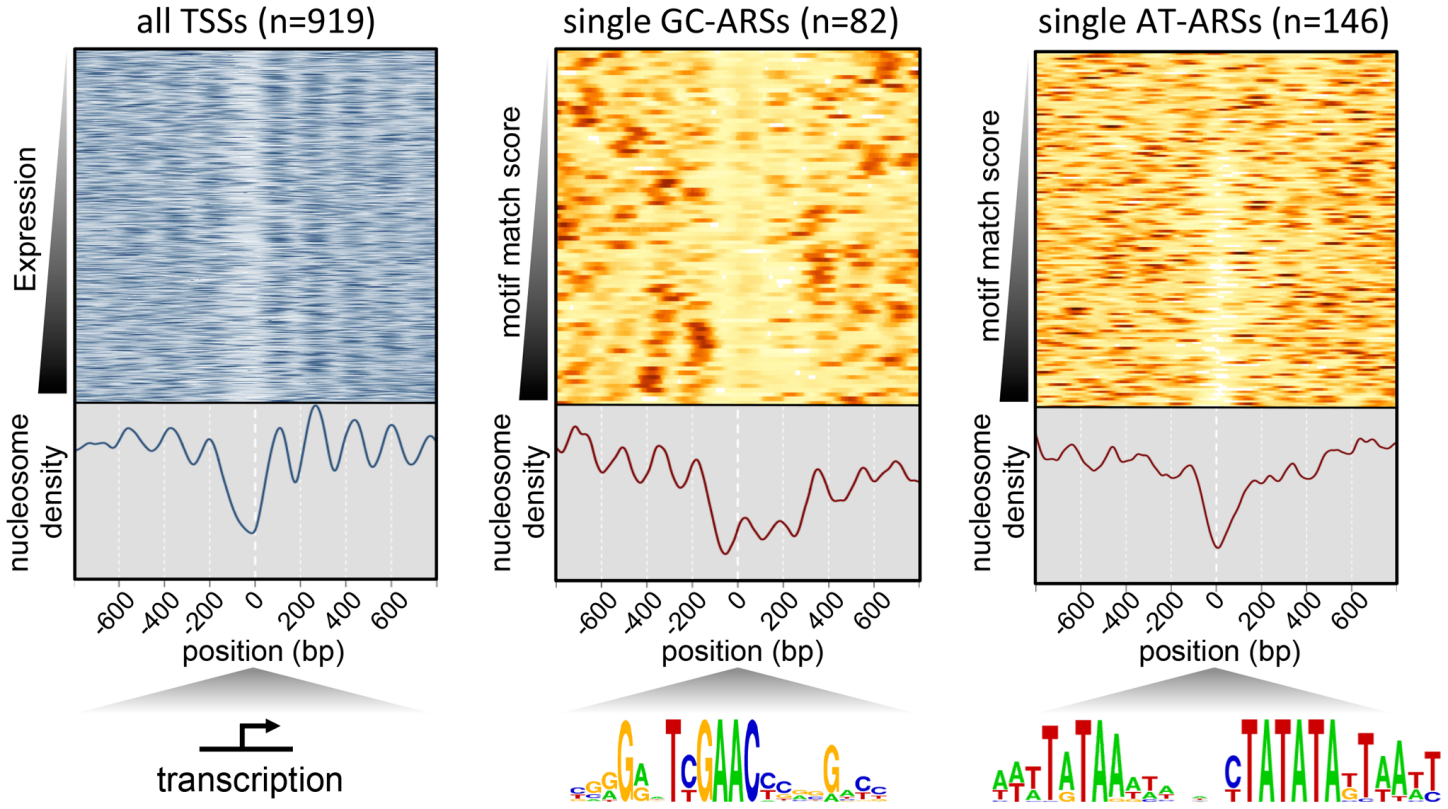
Nucleosome profile of *P. pastoris*. Nucleosome density is plotted for sites centered on all TSSs as a control to test the overall quality of the mapping data (left), non-overlapping GC-ARS sites with a single match to the GC-ACS (middle), or the A/T-rich motif shown in Figure 3C (right). TSS sites are ranked based on expression in the SDEG condition [55]. GC-ARS and AT-ARS sites are ranked by the strength of the best match to the G/C- and the A/T-rich motif respectively.

One common feature of replication origins is a nucleosome depletion region (NDR) close to the site of initiation [13], [14], [26], [30], [51], [52]. To investigate whether this feature holds true for *P. pastoris*, we generated a complete map of nucleosome positions within the *P. pastoris* genome by sequencing genomic DNA digested with micrococcal nuclease [53]. Our results revealed gross nucleosome positioning features similar to those seen in other yeasts, such as an NDR at transcriptional start sites (TSS) followed by regularly positioned nucleosomes within the body of transcripts [54], [55] (Figure 5, Table S7). This result suggests that our experimental methods accurately captured the positions of nucleosomes in this strain. We also detected NDRs at replication origin sites; however, GC-ARS and AT-ARS sites showed striking differences in nucleosome occupancy relative to other budding yeasts [13], [14], [29]. When centered on the GC-ACS, we observed a relative depletion in nucleosome occupancy approximately 40 bp to the 5′ side of the motif (in the TYGAAC orientation). However, unlike other yeast origins where the NDR spans the length of approximately one nucleosome, the *P. pastoris* GC-ARS depletion region spans approximately 450 bp and appears to be excluding three nucleosomes (Figure 5). On the other hand, AT-ARS sites showed a nucleosome depletion region of ∼150 bp in length, a pattern more closely resembling that in other budding yeasts. However, this NDR was not flanked by well-ordered nucleosomes at all AT-ARS sites and suggests either that there are key regulatory differences with other budding yeasts or that not all AT-ARSs use the same sequence determinant for origin firing.

### Genome location and motif sequence identify a class of origins associated with promoters

**Figure 6:**
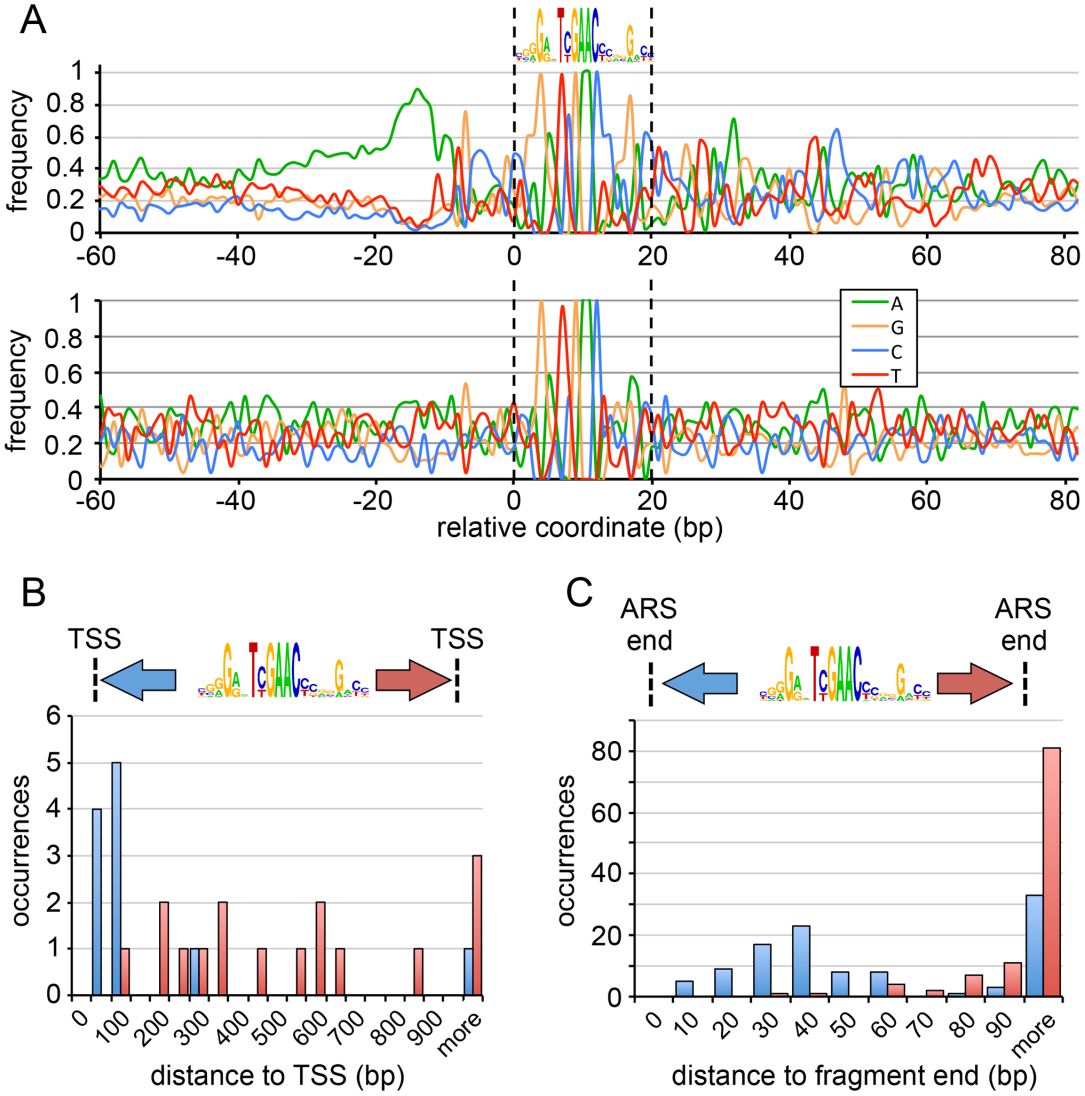
Sequence features of GC-ARSs. (A) Average nucleotide frequencies around 107 GC-ARS sites (top) and twenty-eight non-ARS intergenic occurrences of the GC-ACS (bottom), centered on the best match of the GC-ACS. The nucleotide frequencies are calculated at all flanking regions around the motif independent of whether the flanking region is present in ARS contigs or cores. (B) The distribution of distances between the GC-ACS motif (in the orientation shown) and the TSS for adjacent genes transcribing away from the ARS with available TSS annotations. Distances to the 5′ side of the motif are shown in blue; distances to the 3′ side of the motif are shown in red. (C) The distribution of sequence lengths between the GC-ACS and the end of the inferred functional core region for each GC-ARS. The 5′ distance is indicated in blue; the 3′ distance is indicated in red. Numbers indicate the upper limit of the bin.

The underrepresentation of GC-ARSs in convergently transcribed intergenes (Figure S2) suggests that these elements may be associated with promoters. As in promoters, the NDR near GC-ACS sites is followed by regularly spaced nucleosomes. To test the putative association of the GC-ACS with gene promoters, we searched for this motif in the regulatory motif databases and found that it is a match to one of the motifs annotated as the binding sites of the human Hsf1 [34] heat shock factor (HSF) transcriptional regulator [56] (http://www.factorbook.org/mediawiki/index.php/HSF1). Additionally, when centered on the GC-ACS motif (in the TYGAAC orientation), GC-ARSs show a pronounced poly(dA) region around 10 bp to 35 bp upstream of the motif (Figures 6A and S7A). Notably, this poly(dA) tract is not present near the non-ARS occurrences of this motif and is not required for ARS function (Figure 2). It has been previously shown that such a neighboring poly(dA) region is a conserved feature of Hsf1 binding sites in the *sensu stricto* group of budding yeasts [57], though we note that the TYGAAC portion of the motif does not match the canonical budding yeast HSF motif. To determine whether the GC-ACS is likely to be a binding site for Hsf1 or one of its homologs, we aimed to test whether this motif is overrepresented in promoters of genes likely to be regulated by HSF. We used BLAST to identify homologs of *S. cerevisiae* genes regulated by HSF [58] and filtered the list to include only strong matches (PBLAST E-value<1e-10), resulting in a set of 120 gene homologs. We used the FIMO algorithm to identify significant matches to the GC-ACS within 500 bp regions upstream of all 5037 *P. pastoris* genes. We identified 451 genes that had GC-ACS motifs and 716 genes with matches to the HSF binding site (the Heat Shock Element, HSE [56], [59]), within 500 bp upstream of the start codon. In our set of 120 potential HSF-regulated *P. pastoris* genes, 45 had at least one match to the HSE (hypergeometric test P = 3.1e-11) and 16 genes had GC-ACSs within 500 bp upstream of the start codon (hypergeometric test P = 0.037).

We also used an independent approach to test whether GC-ACS motifs associate with HSE motifs throughout the genome. We mapped separately all occurrences of the GC-ACS and of the HSE. We then assigned to each motif occurrence the nearest annotated gene. There are 5037 annotated genes in *P. pastoris*. From these, 1,188 unique genes were assigned as closest gene to an occurrence of the GC-ACS and 1,236 unique genes were assigned as closest to an HSE. A significant number (524) of unique genes were present in both lists, suggesting an association between GC-ACS and HSE motifs (hypergeometric test P = 4.6e-67). While HSF function in *P. pastoris* has not been studied, these results show an enrichment of GC-ACS motifs in regions likely to be regulated by HSF. Furthermore, the GC-ACS motif is positioned close to TSSs (Figure 6B) and ORF start sites (Figure S8) upstream of the motif suggesting some functional overlap between transcription and early origin firing.

Since the GC-ACS is associated with promoters, it raises the possibility that transcription is required for origin activation. If this possibility were true, then the DNA between the GC-ACS and the TSS may be required for ARS function. Since miniARS-seq screens large numbers of randomly sheared ARS sub-fragments, we were able to test this possibility by determining what sequences flanking the GC-ACS are required for ARS function. Using the full list of inferred functional ARS cores we calculated the length of sequence between the edge of the consensus motif and the edge of the ARS core on either side of the motif (Figure 6C). The distributions of 5′ and 3′ lengths show that several GC-ARSs require <10 bp of sequence on the 5′ of the GC-ACS while more ARS sequence is required on the 3′ side of the motif. In fact, the fragment of ARS-C379 that was used for mutARS-seq (Figure 3A) retained function with only 2 bp of ARS sequence to the 5′ side. Additionally, the twelve wild-type ARS fragments that were tested for activity (Figure 2) all contained <15 bp of sequence to the 5′ of the GC-ACS. The fact that all tested ARSs retained function in the absence of 5′ flanking DNA shows that this region, and the 5′ poly(dA) sequence, are not required for GC-ARS function. While it is possible that transcription can initiate at ectopic sites in the plasmid, these results suggest that transcription *per se* may not be required for GC-ARS function in *P. pastoris*. Consistent with these findings, we have been unable to detect a correlation between expression and replication initiation/timing (data not shown).

The majority of ARSs in budding yeast require sequences on the 3′ side of the ACS (on the T-rich strand) collectively called “B-elements” [38], [42],[60]. Our data show that GC-ARSs also require flanking sequence on the 3′ side of the GC-ACS motif (in the TYGAAC orientation) for ARS function. This result is supported by our mutARS-seq data where we detected a minor region of constrained nucleotides ∼50 bp to the 3′ side of the GC-ACS in ARS-C379 (Figure 3B). The required flanking DNA lies distal to the TSS and may explain the extended nucleosome depletion regions (Figure 5) seen at these loci.

## Discussion

Faithful genome duplication is essential to all living organisms. Like many other cellular processes, DNA replication is primarily regulated at the initiation step. Understanding the regulation of initiation at replication origins is therefore key to understanding how different species replicate their genomes. The extensively studied yeasts *S. cerevisiae* and *S. pombe* have yielded great insights into origin function, but lack several properties exhibited by metazoan origins. For one, metazoan origins have G/C-rich signatures whereas all yeast origin sequence determinants described to date are A/T-rich with the possible exception of fission yeast *S. japonicus*, where GC-rich motifs have been implicated in origin function through sequence analysis. Another key difference between yeast and metazoan origins is the connection between replication initiation and transcription. While promoter-associated origins tend to be early-firing in metazoans, this phenomenon has not been previously described in yeast. These discrepancies limit the value of most yeast species as models for the study of replication origins from higher eukaryotes. A better model would ideally possess the beneficial characteristics of yeast (genetic and molecular tools) while also recapitulating more of the traits displayed by metazoans.

In this study we generated a comprehensive profile of replication origins in *P. pastoris*, a budding yeast that is very distantly related to both the *S. cerevisiae* and *S. pombe* yeasts [61]. This methylotrophic budding yeast has traditionally been utilized as an industrial organism valued for its ability to convert methanol to biomass and for its ability to produce and secrete recombinant proteins in high yields [33]. An early study showed that two native *P. pastoris* ARSs did not function in *S. cerevisiae*, suggesting key mechanistic differences in replication initiation between the two species [37]. We identified 311 ARSs in *P. pastoris* and were able to delineate the essential functional regions to <200 bp in most cases. As in other budding yeasts we found PpARSs to reside predominantly in intergenic regions. However, unlike other studied yeasts, *P. pastoris* displayed a conserved G/C-rich motif (GC-ACS) in approximately 35% of its ARSs. In fact, almost all strong intergenic matches to this motif were isolated in our ARS screen, suggesting a causal role for this motif in origin function. We were unable to detect a strong conserved motif within the other origins (AT-ARSs). It is possible that the AT-ARSs function with an ill-defined sequence determinant similar to those seen in *S. pombe* and *L. kluyveri* [22], [28] or that the sequence required for AT-ARS function is innately elusive to traditional alignment-based methods due to its nucleotide composition.

To identify experimentally the nucleotides required for ARS function, we used mutARS-seq, a massively parallel approach that allows simultaneous measurement of the effects of all mutations on the function of an ARS [38]. This approach showed that the GC-ACS is indeed required for GC-ARS function (Figure 3B). Notably, the GC-ACS was the most constrained element within the ARS tested, suggesting that this motif is the primary element used for ARS function and not a supporting element akin to *S. cerevisiae* “B-elements”. The fact that the GC-ACS motif retains function within different plasmid contexts supports this hypothesis. The mutARS-seq experiment on ARS-A2772, an AT-ARS, revealed a very different region of functional constraint (Figure 3C). A repetitive A/T-rich element was required for the function of this ARS. Other than its general A/T-richness, this element is significantly different from all previously identified ACS elements. Similarly to the GC-ACS, this motif is also the only strong region of functional constraint within the ARS and functions within different plasmid contexts, suggesting that it is a primary ARS element. While it is tempting to speculate that both of these motifs act as ORC binding sites (or in some other way recruit relevant protein factors), we have no direct evidence to this effect. To our knowledge *P. pastoris* is the first organism that simultaneously uses such diverse sequences as ARS elements.

The dynamics of replication in this species showed a surprising difference in replication timing between GC-ARSs and AT-ARSs (Figure 4). While both types of origins exist within replication peaks, as a class, GC-ARS sites replicate significantly earlier and/or more efficiently than AT-ARS sites—although there are individual exceptions to this general categorization (Figure 4B). Our data also show that while the timing/efficiency of AT-ARS benefits from clustering with other ARSs, GC-ARSs are not affected by clustering, suggesting that they are operating at maximal initiation potential. While it is not yet clear how such a hierarchy of replication timing is achieved mechanistically, in metazoan cells promoter-associated origins fire earlier than the others and this difference is usually attributed to increased chromatin accessibility at transcription start sites [1]. Our findings are consistent with the difference in timing being associated with differences in chromatin structure. We assayed global positioning of nucleosomes in *P. pastoris* by sequencing mononucleosomal DNA from MNase-treated chromatin. The results of this experiment (Figure 5) showed an atypical pattern of nucleosome depletion at GC-ARSs that resembles the depletion pattern seen at TSSs, but with two additional nucleosomes depleted upstream of the TSS. In contrast, nucleosome depletion at AT-ARSs resembles the *S. cerevisiae* ARS pattern with a single nucleosome depleted close to the location of the A/T-rich functional element. It should also be noted that while the A/T-rich motif identified by mutARS-seq is essential for the function of ARS-A2772, it is possible that other AT-ARSs use other elements. This possibility is supported by the fact that many AT-ARSs do not have strong matches to the motif generated from the mutARS-seq data despite showing a nucleosome depletion region at the site of best match.

Combined, our findings suggest that *P. pastoris* can utilize at least two distinct sequences for origin selection and activation. One group of origins is A/T-rich and their replication times are distributed across S phase. The other type of origin is G/C-rich, disproportionally early replicating, and shows a close association with transcription start sites, properties usually associated with metazoan origins. In fact, the conserved motif required for GC-ARS firing is a very close match to the binding site of the human Hsf1 transcriptional activator [34]. Additionally, we have detected a statistical association between GC-ACS motifs and genes likely to be regulated by Hsf1 or its homologs. While the mechanistic nature of GC-ARS function will require additional investigation, our data could suggest that the Hsf1 binding site in *P. pastoris* is capable of recruiting either directly or indirectly the replication initiation machinery. Our data also suggest that transcription *per se* may not be required for GC-ARS function (Figure 6C), as sequences between the GC-ACS and transcription start sites are not required for ARS function, but are likely to be required for transcription. Consistent with this model, we have not been able to detect a correlation between gene expression and replication timing, but this lack of correlation may also be due to a combination of subtle regulation patterns and scarcity of available expression data. It is worth noting that the GC-ACS motif does not match the well-defined *S. cerevisiae* Hsf1 binding site that has the sequence structure TTCTAGAAnnTTCT [62] and is often represented as three evenly-spaced trinucleotides TTCnnGAAnnTTC [59]. However, Hsf1 is known to directly regulate genes lacking this motif, suggesting an ability to interact with diverse sequences [58]. Barring a mis-annotation, it is possible that in *P. pastoris* at least one of the four Hsf1 homologs is able to interact with and recruit ORC whereas the single Hsf1 protein in *S. cerevisiae* cannot bind to this atypical motif and thus relies exclusively on A/T-rich ARSs. This hypothesis would imply that the ability to use G/C-rich motifs for replication initiation is an ancestral trait that was lost in the lineage leading to the *Saccharomyces*, *Lachancea*, and *Kluyveromyces* clades. Whether other budding yeasts can utilize G/C-rich sites for initiation is not yet known. Alternatively, since a connection between Hsf1 and replication initiation has not yet been described, it is possible that this novel function is specific to the *Pichia* (*Komagataella*) genus, or perhaps only *P. pastoris*. Another observation that points to this motif being used for multiple functions is that a G/C-rich motif constructed from mutARS-seq data (Figures 3B and S7B) is less information-rich than the motif obtained from alignment (contrary to the case of the A/T-rich motif which is difficult to produce by alignment, but is very obvious in the mutARS-seq data). While the optimal bases within the mutARS-seq data perfectly match the alignment-based motif, the cost of changing to a sub-optimal nucleotide is lower at most positions than the alignment-based motif would suggest. This observation can be explained by hypothesizing that this GC-motif is used for both origin activity as well as transcriptional regulation. If transcriptional regulation of the genes affected by this motif is evolutionarily more constrained than is ARS activity, then we would expect that the G/C-rich motifs would be selected upon primarily for their regulatory function.

Additionally, it is possible that GC-ACS motifs act as enhancer elements to other, potentially A/T-rich primary elements. Transcription factors such as Fkh1, Abf1, and Mcm1 have been previously shown to enhance origin activity in *S. cerevisiae* [10]–[12]. This model would argue that the G/C-rich motif does not act as a primary site of initiation, but enables nearby dormant elements to initiate DNA replication possibly through the chromatin-modifying activity of Hsf1. However, the fact that approximately one-third of all active origins have the same G/C-rich motif and that almost all intergenic occurrences of this motif are in ARSs is very different from what has been previously observed in other yeast models where connections between ARSs and transcription factors are much less obvious.

In addition to elucidating the features of replication dynamics, our data offer useful tools and data resources for this industrially important yeast. We anticipate that our nucleosome position map will be useful for studies of chromatin and gene expression, especially when combined with transcriptome data [55], [63]. More practically, replication origins are regulators of genome duplication and cell cycle progression, and are essential for episomal plasmid maintenance [64]. Current episomal vectors used in *P. pastoris* contain the original PARS1 (ARS-B413 in our data), an ARS discovered almost three decades ago [37], [65]. Our data show that PARS1 is one of the less efficient AT-ARSs (Figure S6) [64], suggesting that using a different ARS may result in improvements in plasmid stability. Previously, we used mutARS-seq data to optimize ARS function in *S. cerevisiae* [38] and this approach can potentially be used to further improve plasmid maintenance in *P. pastoris*, facilitating strain engineering efforts in this system.

## Materials and Methods

### Strains, plasmids, and reagents

The *P. pastoris* strain used in these studies was JC308 (James Cregg), a *ura3* auxotroph of the GS115 background strain. All yeast growth was performed at 30°C; all bacterial growth was performed at 37°C. The plasmid vectors used in this study were previously described [38]. All *E. coli* work was done using Alpha-Select Gold Efficiency competent cells (Bioline). All enzymes used were from New England Biolabs unless otherwise noted. Primers were purchased from IDT unless otherwise noted. PCR purification and purification of digested plasmids was done using the DNA Clean and Concentrator-5 Kit (Zymo Research). Plasmid DNA was purified using the Wizard Plus SV Miniprep Kit (Promega).

### ARS-seq and miniARS-seq

ARS-seq and miniARS-seq screens were performed largely as described [38]. *P. pastoris* genomic DNA was isolated from cells grown in YPD using a phenol/chloroform bead-disruption method followed by ultracentrifugation in a CsCl gradient (to remove mitochondrial DNA) followed by EtOH precipitation. Genomic DNA was fragmented and ligated as described [38]. Cloning efficiencies of resultant libraries were verified by colony PCR and *P. pastoris* cells were transformed with libraries using a custom lithium acetate protocol as follows. To make competent cells yeast were grown in YPG medium (10 g/L yeast extract, 20 g/L Peptone, 3% v/v glycerol) until OD_600_ density of 1. Cells from 1L of culture were spun down, rinsed and resuspended in 10 mL of TE/LiOAc (10 mM Tris-HCl, 1 mM EDTA, 100 mM lithium acetate). Cell suspensions were incubated at 30°C with shaking for 30 minutes, dispensed into 100 µL aliquots and frozen at −80°C. For transformations competent cells were thawed at room temperature, mixed with 1–5 µg of plasmid DNA, 600 µL of “two-step” transformation buffer (40% polyethylene glycol-4000, 100 mM LiOAc, 10 mM Tris-HCl, 1 mM EDTA, 12 mM DTT, 0.12 mg/mL fish sperm carrier DNA) and incubated at 30°C with gentle rotation for 30 minutes. The cell mixture was then heat-shocked at 42°C for 30 minutes and plated. Cells were grown for five days, replica-plated, and grown for three more days before cells were pooled for plasmid extraction. DNA shearing for miniARS-seq, plasmid recovery from yeast, and Illumina sequencing were performed as described [38].

### ARS-seq and miniARS-seq sequence analysis

Illumina paired end sequencing reads were uniquely mapped to the GS115 genome [31] using Bowtie version 0.12.7. Custom Python scripts were used to detect relevant restriction sites at the ends of all mapped fragments that were extended to remove truncation products. Overlapping fragments were assembled into contigs. Contigs that had a combined read-depth of 1 were removed from the dataset. Cases where multiple discontinuous contigs were joined by overlapping fragments were manually resolved based on read depth. To maximize miniARS-seq data recovery, 101 bp paired end reads were mapped in full and unmapped reads were trimmed to 50 bp and mapped again. Resulting fragments with read depth >1 were assembled into contigs and contigs consisting of fewer than three unique fragments were removed. Both ARS-seq and miniARS-seq fragments were used to delineate minimal overlapping regions (“inferred functional cores”). To prevent data loss, cores that were <150 bp in length were extended bi-directionally to a final length of 150 bp.

### mutARS-seq

mutARS-seq was performed largely as described [38]. Mutagenized oligos of ARS-C379 and ARS-A2772 were synthesized by Trilink Biotechnologies. The resulting libraries contained 24,000—40,000 ARS inserts. Yeast were transformed with mutagenized libraries as described above in two biological replicate pools each containing ∼100,000 transformed colonies. After five days of growth on selective agar plates, colonies were pooled and inoculated into 1 L cultures of liquid selective medium. Cultures were grown for 36 hours with periodic dilution to prevent saturation. Samples were taken at 0, 12, 24, and 36 hours. Sequencing data were analyzed using the Enrich software package [66]. For maximum separation averaged data from the 36-hour samples are shown in Figure 3. To create a position-weighted matrix from mutARS-seq data, the enrichment ratio values within the constrained region were converted into relative allele frequencies after an arbitrary cutoff minimum of 0.2 was applied. Logo images were generated using Weblogo software [67].

### Site-directed mutagenesis

ARS sequences bearing mutations (Table S4) were ordered as custom designed double stranded gBlock DNA fragments (Integrated DNA Technologies). The gBlocks were used as PCR templates to amplify the mutant alleles prior to cloning. Wild type ARS alleles were PCR amplified from the gDNA of the parent strain (JC308).

### Conserved motif analysis

The MEME de novo motif discovery tool [44] was applied to identify conserved motifs within the entire set of PpARSs using the 5th order Markov background model and the entire set of *P. pastoris* intergenic sequences. Both MAST [68] and FIMO [69] programs from the MEME suite were used to map motif occurrences within different sets of ARS sequences.

### 2D gel analysis

A 1 L culture of *P. pastoris* was grown to early log phase in YEPD and harvested for genomic DNA isolation [70]. Approximately 8 µg of DNA was cleaved with NcoI or StuI to release genomic fragments of 4.575 kb or 4.043 kb containing ARS-C379 or the ARS-A2772, respectively. Replication intermediates were separated on a first dimension gel of 0.4% ME agarose in 1×TBE for 20 hours at 1 V/cm. Lanes for the second dimension gel were sliced from the gel and encased in a second gel of 0.9% ME agarose in 1×TBE with 0.3 µg/ml. Electrophoresis for the second dimension was carried out for 4.5 hours at 5.5 V/cm at 4°C. The genomic fragments were detected on Southern blots using ^32^P-dATP labeled PCR probes.

### Replication timing measurements

Replication timing experiments were performed largely as described [48]. Exponentially growing (in YPD medium) *P. pastoris* cells were subjected to flow sorting using standard techniques on a BD FACsAria II cell-sorter. The purity of each sorted sample was determined to be ∼95%. Genomic DNA from 1.5–2 million G1 and S-phase cells was isolated using the YeaStar Genomic DNA Kit (Zymo Research). Randomly fragmented sequencing libraries were prepared using the Nextera DNA Sample Preparation Kit (Illumina) [71]. Approximately 29 million 50 bp reads were recovered for each sample of each replicate. More than 90% of the reads in all samples were mapped to the *P. pastoris* GS115 reference genome and ∼1% of the reads in each sample were removed due to multiple mapping sites. After processing, 25–27 million reads were assigned to 1 kb bins across the genome resulting in average count-depth of 2936 reads/bin for G1 sample of replicate 1, 2796 reads/bin for G1 sample of replicate 2, 2843 reads/bin for S sample of replicate 1, and 2913 reads/bin for S sample of replicate 2. Reads were mapped using Bowtie and custom scripts were used to generate replication timing profiles as described [48]. The total number of reads for each replicate was equalized in each sample and a ratio of S/G1 reads was calculated for each replicate. These ratios were multiplied by 1.5 to account for the fact that the average cell in the middle of S-phase will have replicated half of its DNA. We fitted a loess curve to the mean of the two replicate ratio measurements, then found peaks along this curve using the turnpoints() function from the R package, pastecs. The resulting curves were normalized to a baseline value of 1.

### Nucleosome mapping

Nucleosome positions were mapped similarly to the method described [53]. Two colonies were grown in 400 mL of YPD media until an OD_600_ of 1 and then cross-linked with formaldehyde. The two samples were bead disrupted in 10 mM Tris-HCl pH 8.0 with 1 mM CaCl_2_. Visually lysed samples were then MNase digested for 30 minutes at increasing concentrations of MNase. Cross-links were removed by overnight incubation at 65°C followed by DNA extraction with phenol/chloroform. Extracted DNA was separated using a 2% agarose gel to visualize the mononucleosome enriched band. DNA corresponding to ∼150 bp was then extracted and sequenced using the Illumina HiSeq platform. The samples were divided in half to provide technical replicates.

### Accession numbers

All sequencing data presented are available from the National Center for Biotechnology Information Sequence Read Archive (ARS-seq - SRP031643; miniARS-seq - SRP031646; mutARSseq - SRP031760; replication timing - SRP031759; nucleosome mapping - SRP031651).

## Supporting Information

Figure S1 Summary of ARS-seq and miniARS-seq results. (A) ARS fragment length distributions. The distribution of lengths of unique ARS-seq and miniARS-seq inserts as shown in Tables S1 and S2. (B) ARS-seq read depth distributions. The combined read depths for all ARSs identified by ARS-seq are shown in red (AT-ARSs) and blue (GC-ARSs). The combined read depths for each ARS are available in Table S3.
(PDF)

Figure S2 The direction of genes flanking intergenic ARSs. Intergenic regions >1 bp in length [31] are grouped based on the direction of transcription of the pair of genes flanking the intergenic space. Numbers of intergenes falling into convergent (left), divergent (middle), and tandem (right) orientations are shown (All intergenes). The numbers of GC-ARS or AT-ARS containing intergenes in different orientation groups are shown (GC-ARSs and AT-ARSs respectively). Numbers in parentheses refer to the number of intergenes expected in the given group assuming a random distribution.
(PDF)

Figure S3 ARS-C379 mutARS-seq data during competitive growth. Data processed as described (Methods) is shown as the average of two replicates for 12-, 24-, and 36-hour timepoints normalized against the same input sample. Data are plotted on the same y-axis scale to aid visual comparison. Scatterplots show correlations between replicates of the same timepoint samples (lower panels).
(PDF)

Figure S4 ARS-A2772 mutARS-seq data during competitive growth. Data processed as described (Methods) is shown as the average of two replicates for 12-, 24-, and 36-hour timepoints normalized against the same input sample. Data are plotted on the same y-axis scale to aid visual comparison. Scatterplots show correlations between replicates of the same timepoint samples (lower panels).
(PDF)

Figure S5 Comparisons of mutARS-seq data during competitive growth. Averaged mutARS-seq data from 12-, 24-, and 36-hour timepoints are plotted as scatterplots.
(PDF)

Figure S6 Replication profiles of all *P. pastoris* chromosomes. Replication timing profiles were computed as discussed (Figure 4) and are shown for all four *P. pastoris* chromosomes. Un-smoothed ratio data for one of the replicates is shown in grey. Locations of GC-ARSs and AT-ARSs are indicated by open and shaded circles respectively.
(PDF)

Figure S7 Comparison of GC-ARS motifs. (A) The ACS motif identified from 107 GC-ARSs when the motif length is forced to be 50 bp. (B) The motif obtained from mutARS-seq of ARS-C379 using a procedure identical to the one used to obtain the AT-rich motif in Figure 3C.
(PDF)

Figure S8 Distance between GC-ACS and flanking ATGs. For all instances where the GC-ARS is found adjacent to the 5′ end of a gene, we calculated the distance between the start ATG codon of the ORF and the closest edge of the GC-ACS match. The distributions of ATG-to-ACS distances are plotted as histograms based on the direction relative to the ACS.
(PDF)

Table S1 List of ARS-seq fragments. “fragment_name”: a unique identifier for each fragment consisting of the fragment's endpoint coordinates. “chrom”: chromosome containing the fragment. “start”: the leftmost coordinate of the fragment. “end”: the rightmost coordinate of the fragment. “rd”: the read depth of the fragment. “restriction_fragment”: the restriction enzyme digest which made this fragment, “unk” = cannot assign to a single site.
(XLSX)

Table S2 List of miniARS-seq fragments. “miniARS_fragment_name: a unique identifier for each fragment consisting of the fragment's endpoint coordinates. “chrom”: chromosome containing the fragment. “start”: the leftmost coordinate of the fragment. “end”: the rightmost coordinate of the fragment. “rd”: the read depth of the fragment. “ARSseq_contig_name”: the parent ARS-seq contig of this sub-fragment. “contig_count”: the total number of miniARS fragments assigned to this parent contig.
(XLSX)

Table S3 Compiled list of PpARSs. “ARS_name”: The systematic name of the ARS, based on chromosomal location. “contig_name”: a unique identifier for each fragment consisting of the fragment's endpoint coordinates. “chrom”: chromosome containing the fragment. “start”: the leftmost coordinate of the fragment. “end”: the rightmost coordinate of the fragment. “combined_rd”: the combined read depth of all ARS-seq fragments in this contig. “fragment_count”: the number of ARS-seq fragments assigned to this contig. “ARSseq_core_start”: leftmost coordinate of the inferred functional core of this ARS. “ARSseq_core_end”: rightmost coordinate of the inferred functional core of this ARS. “ARSseq_core_len”: length of the inferred functional core of this ARS. “miniARS_core_start”: leftmost coordinate of the inferred functional core of this miniARS (no value indicates this ARS was not represented in the final miniARS dataset). “miniARS_core_end”: leftmost coordinate of the inferred functional core of this miniARS. “miniARS_core_len”: length of the inferred functional core of this miniARS. “manually_confirmed”: has some fragment of this ARS been manually validated?
(XLSX)

Table S4 Manual validation results. “fragment_name”: the fragment orARS tested. “chrom”: chromosome containing the fragment. “start”: the leftmost coordinate of the fragment. “end”: the rightmost coordinate of the fragment. “length”: the length of the fragment tested. “combined_rd”: the read depth of the ARS (“manual” indicates that the ARS was not present in screen data).
(XLSX)

Table S5 Replication timing results. “bin_name”: a unique identifier for each 1 kb bin within the genome. “chrom”: chromosome containing the bin. “coord_kb”: the value of the first position coordinate of the corresponding bin. “replicate1_ratio”: the relative replication ratio for biological replicate 1. “replicate1_ratio”: the relative replication ratio for biological replicate 2. “normalized_ratio”: averaged, smoothed, and normalized replication ratio as described.
(XLSX)

Table S6 Locations of replication timing peaks. “bin_name”: the unique identifier of the 1 kb bin containing the center of the peak. “chrom”: chromosome containing the bin “coord_kb”: the value of the first position coordinate of the corresponding bin.
(XLSX)

Table S7 Nucleosome position values. “chrom”: chromosome. “coord”: coordinate within the chromosome. “P1_A”: nucleosome density in biological replicate 1, technical replicate 1. “P1_B”: nucleosome density in biological replicate 1, technical replicate 2. “P2_A”: nucleosome density in biological replicate 2, technical replicate 1. “P2_B”: nucleosome density in biological replicate 2, technical replicate 2. “mean”: the mean value of the four samples.
(ZIP)

## References

[1] Méchali M (2010) Eukaryotic DNA replication origins: many choices for appropriate answers. Nat Rev Mol Cell Biol 11: 728–738 doi:10.1038/nrm2976 20861881 10.1038/nrm2976

[2] Eaton ML, Prinz JA, MacAlpine HK, Tretyakov G, Kharchenko PV, et al (2011) Chromatin signatures of the *Drosophila* replication program. Genome Res 21: 164–174 doi:10.1101/gr.116038.110 21177973 10.1101/gr.116038.110 PMC3032920

[3] Dellino GI, Cittaro D, Piccioni R, Luzi L, Banfi S, et al (2013) Genome-wide mapping of human DNA-replication origins: Levels of transcription at ORC1 sites regulate origin selection and replication timing. Genome Res 23: 1–11 doi:10.1101/gr.142331.112 23187890 10.1101/gr.142331.112 PMC3530669

[4] Costas C, la Paz Sanchez de M, Stroud H, Yu Y, Oliveros JC, et al (2011) Genome-wide mapping of *Arabidopsis thaliana* origins of DNA replication and their associated epigenetic marks. Nat Struct Mol Biol 18: 395–400 doi:10.1038/nsmb.1988 21297636 10.1038/nsmb.1988 PMC3079358

[5] Hansen RS, Thomas S, Sandstrom R, Canfield TK, Thurman RE, et al (2010) Sequencing newly replicated DNA reveals widespread plasticity in human replication timing. Proc Natl Acad Sci USA 107: 139–144 doi:10.1073/pnas.0912402107 19966280 10.1073/pnas.0912402107 PMC2806781

[6] Méchali M, Yoshida K, Coulombe P, Pasero P (2013) Genetic and epigenetic determinants of DNA replication origins, position and activation. Curr Opin Genet Dev 23: 124–131 doi:10.1016/j.gde.2013.02.010 23541525 10.1016/j.gde.2013.02.010

[7] Cayrou C, Coulombe P, Vigneron A, Stanojcic S, Ganier O, et al (2011) Genome-scale analysis of metazoan replication origins reveals their organization in specific but flexible sites defined by conserved features. Genome Res 21: 1438–1449 doi:10.1101/gr.121830.111 21750104 10.1101/gr.121830.111 PMC3166829

[8] Stinchcomb DT, Struhl K, Davis RW (1979) Isolation and characterisation of a yeast chromosomal replicator. Nature 282: 39–43. 388229 10.1038/282039a0

[9] Bell SP, Dutta A (2002) DNA replication in eukaryotic cells. Annu Rev Biochem 71: 333–374 doi:10.1146/annurev.biochem.71.110601.135425 12045100 10.1146/annurev.biochem.71.110601.135425

[10] Chang VK, Donato JJ, Chan CS, Tye BK (2004) Mcm1 promotes replication initiation by binding specific elements at replication origins. Mol Cell Biol 24: 6514–6524 doi:10.1128/MCB.24.14.6514-6524.2004 15226450 10.1128/MCB.24.14.6514-6524.2004 PMC434236

[11] Walker SS, Francesconi SC, Tye BK, Eisenberg S (1989) The OBF1 protein and its DNA-binding site are important for the function of an autonomously replicating sequence in *Saccharomyces cerevisiae* . Mol Cell Biol 9: 2914–2921. 2674674 10.1128/mcb.9.7.2914-2921.1989 PMC362758

[12] Knott SRV, Peace JM, Ostrow AZ, Gan Y, Rex AE, et al (2012) Forkhead Transcription Factors Establish Origin Timing and Long-Range Clustering in *S. cerevisiae* . Cell 148: 99–111 doi:10.1016/j.cell.2011.12.012 22265405 10.1016/j.cell.2011.12.012 PMC3266545

[13] Eaton ML, Galani K, Kang S, Bell SP, MacAlpine DM (2010) Conserved nucleosome positioning defines replication origins. Genes Dev 24: 748–753 doi:10.1101/gad.1913210 20351051 10.1101/gad.1913210 PMC2854390

[14] Berbenetz NM, Nislow C, Brown GW (2010) Diversity of eukaryotic DNA replication origins revealed by genome-wide analysis of chromatin structure. PLoS Genet 6: e1001092 doi:10.1371/journal.pgen.1001092 20824081 10.1371/journal.pgen.1001092 PMC2932696

[15] Lin S, Kowalski D (1997) Functional equivalency and diversity of cis-acting elements among yeast replication origins. Mol Cell Biol 17: 5473–5484. 9271423 10.1128/mcb.17.9.5473 PMC232396

[16] Donaldson AD, Raghuraman MK, Friedman KL, Cross FR, Brewer BJ, et al (1998) *CLB5*-dependent activation of late replication origins in *S. cerevisiae* . Mol Cell 2: 173–182. 9734354 10.1016/s1097-2765(00)80127-6

[17] Koren A, Tsai H-J, Tirosh I, Burrack LS, Barkai N, et al (2010) Epigenetically-inherited centromere and neocentromere DNA replicates earliest in S-phase. PLoS Genet 6: e1001068 doi:10.1371/journal.pgen.1001068 20808889 10.1371/journal.pgen.1001068 PMC2924309

[18] Mantiero D, Mackenzie A, Donaldson A, Zegerman P (2011) Limiting replication initiation factors execute the temporal programme of origin firing in budding yeast. EMBO J 30: 4805–4814 doi:10.1038/emboj.2011.404 22081107 10.1038/emboj.2011.404 PMC3243606

[19] Bechhoefer J, Rhind N (2012) Replication timing and its emergence from stochastic processes. Trends Genet 28: 374–381 doi:10.1016/j.tig.2012.03.011 22520729 10.1016/j.tig.2012.03.011 PMC3401328

[20] de Moura APS, Retkute R, Hawkins M, Nieduszynski CA (2010) Mathematical modelling of whole chromosome replication. Nucleic Acids Res 38: 5623–5633 doi:10.1093/nar/gkq343 20457753 10.1093/nar/gkq343 PMC2943597

[21] Chuang RY, Kelly TJ (1999) The fission yeast homologue of Orc4p binds to replication origin DNA via multiple AT-hooks. Proc Natl Acad Sci USA 96: 2656–2661. 10077566 10.1073/pnas.96.6.2656 PMC15824

[22] Dai J, Chuang R-Y, Kelly TJ (2005) DNA replication origins in the *Schizosaccharomyces pombe* genome. Proc Natl Acad Sci USA 102: 337–342 doi:10.1073/pnas.0408811102 15623550 10.1073/pnas.0408811102 PMC539312

[23] Patel PK, Arcangioli B, Baker SP, Bensimon A, Rhind N (2006) DNA replication origins fire stochastically in fission yeast. Mol Biol Cell 17: 308–316 doi:10.1091/mbc.E05-07-0657 16251353 10.1091/mbc.E05-07-0657 PMC1345668

[24] Ryba T, Hiratani I, Sasaki T, Battaglia D, Kulik M, et al (2011) Replication timing: a fingerprint for cell identity and pluripotency. PLoS Comput Biol 7: e1002225 doi:10.1371/journal.pcbi.1002225 22028635 10.1371/journal.pcbi.1002225 PMC3197641

[25] Delgado S, Gómez M, Bird A, Antequera F (1998) Initiation of DNA replication at CpG islands in mammalian chromosomes. EMBO J 17: 2426–2435 doi:10.1093/emboj/17.8.2426 9545253 10.1093/emboj/17.8.2426 PMC1170585

[26] MacAlpine HK, Gordân R, Powell SK, Hartemink AJ, MacAlpine DM (2010) *Drosophila* ORC localizes to open chromatin and marks sites of cohesin complex loading. Genome Res 20: 201–211 doi:10.1101/gr.097873.109 19996087 10.1101/gr.097873.109 PMC2813476

[27] Liachko I, Bhaskar A, Lee C, Chung SCC, Tye B-K, et al (2010) A comprehensive genome-wide map of autonomously replicating sequences in a naive genome. PLoS Genet 6: e1000946 doi:10.1371/journal.pgen.1000946 20485513 10.1371/journal.pgen.1000946 PMC2869322

[28] Liachko I, Tanaka E, Cox K, Chung SCC, Yang L, et al (2011) Novel features of ARS selection in budding yeast *Lachancea kluyveri* . BMC Genomics 12: 633 doi:10.1186/1471-2164-12-633 22204614 10.1186/1471-2164-12-633 PMC3306766

[29] Di Rienzi SC, Lindstrom KC, Mann T, Noble WS, Raghuraman MK, et al (2012) Maintaining replication origins in the face of genomic change. Genome Res 22: 1940–1952 doi:10.1101/gr.138248.112 22665441 10.1101/gr.138248.112 PMC3460189

[30] Xu J, Yanagisawa Y, Tsankov AM, Hart C, Aoki K, et al (2012) Genome-wide identification and characterization of replication origins by deep sequencing. Genome Biol 13: R27 doi:10.1186/gb-2012-13-4-r27 22531001 10.1186/gb-2012-13-4-r27 PMC3446301

[31] De Schutter K, Lin Y-C, Tiels P, Van Hecke A, Glinka S, et al (2009) Genome sequence of the recombinant protein production host *Pichia pastoris* . Nat Biotechnol 27: 561–566 doi:10.1038/nbt.1544 19465926 10.1038/nbt.1544

[32] Kurtzman CP (2009) Biotechnological strains of *Komagataella (Pichia) pastoris* are *Komagataella phaffii* as determined from multigene sequence analysis. J Ind Microbiol Biotechnol 36: 1435–1438 doi:10.1007/s10295-009-0638-4 19760441 10.1007/s10295-009-0638-4

[33] Macauley-Patrick S, Fazenda ML, McNeil B, Harvey LM (2005) Heterologous protein production using the *Pichia pastoris* expression system. Yeast 22: 249–270 doi:10.1002/yea.1208 15704221 10.1002/yea.1208

[34] Anckar J, Sistonen L (2011) Regulation of HSF1 function in the heat stress response: implications in aging and disease. Annu Rev Biochem 80: 1089–1115 doi:10.1146/annurev-biochem-060809-095203 21417720 10.1146/annurev-biochem-060809-095203

[35] Chan CS, Tye BK (1980) Autonomously replicating sequences in *Saccharomyces cerevisiae* . Proc Natl Acad Sci USA 77: 6329–6333. 7005897 10.1073/pnas.77.11.6329 PMC350277

[36] Tanaka S, Tanaka Y, Isono K (1996) Systematic mapping of autonomously replicating sequences on chromosome V of *Saccharomyces cerevisiae* using a novel strategy. Yeast 12: 101–113 doi:;10.1002/(SICI)1097-0061(199602)12:2<101::AID-YEA885>3.0.CO;2-2 8686374 10.1002/(sici)1097-0061(199602)12:2<101::aid-yea885>3.0.co;2-2

[37] Cregg JM, Barringer KJ, Hessler AY, Madden KR (1985) *Pichia pastoris* as a host system for transformations. Mol Cell Biol 5: 3376–3385. 3915774 10.1128/mcb.5.12.3376-3385.1985 PMC369166

[38] Liachko I, Youngblood RA, Keich U, Dunham MJ (2013) High-resolution mapping, characterization, and optimization of autonomously replicating sequences in yeast. Genome Res 23: 698–704 doi:10.1101/gr.144659.112 23241746 10.1101/gr.144659.112 PMC3613586

[39] Keich U, Gao H, Garretson JS, Bhaskar A, Liachko I, et al (2008) Computational detection of significant variation in binding affinity across two sets of sequences with application to the analysis of replication origins in yeast. BMC Bioinformatics 9: 372 doi:10.1186/1471-2105-9-372 18786274 10.1186/1471-2105-9-372 PMC2566582

[40] Ng P, Keich U (2008) GIMSAN: a Gibbs motif finder with significance analysis. Bioinformatics 24: 2256–2257 doi:10.1093/bioinformatics/btn408 18703586 10.1093/bioinformatics/btn408

[41] Breier AM, Chatterji S, Cozzarelli NR (2004) Prediction of *Saccharomyces cerevisiae* replication origins. Genome Biol 5: R22 doi:10.1186/gb-2004-5-4-r22 15059255 10.1186/gb-2004-5-4-r22 PMC395781

[42] Nieduszynski CA, Knox Y, Donaldson AD (2006) Genome-wide identification of replication origins in yeast by comparative genomics. Genes Dev 20: 1874–1879 doi:10.1101/gad.385306 16847347 10.1101/gad.385306 PMC1522085

[43] Bhaskar A, Keich U (2010) Confidently estimating the number of DNA replication origins. Stat Appl Genet Mol Biol 9: Article28 doi:10.2202/1544-6115.1544 20678076 10.2202/1544-6115.1544

[44] Bailey TL, Elkan C (1994) Fitting a mixture model by expectation maximization to discover motifs in biopolymers. Proc Int Conf Intell Syst Mol Biol 2: 28–36. 7584402

[45] Brewer BJ, Fangman WL (1987) The localization of replication origins on ARS plasmids in *S. cerevisiae* . Cell 51: 463–471. 2822257 10.1016/0092-8674(87)90642-8

[46] Fowler DM, Araya CL, Fleishman SJ, Kellogg EH, Stephany JJ, et al (2010) High-resolution mapping of protein sequence-function relationships. Nat Methods 7: 741–746 doi:10.1038/nmeth.1492 20711194 10.1038/nmeth.1492 PMC2938879

[47] Patwardhan RP, Lee C, Litvin O, Young DL, Pe'er D, et al (2009) High-resolution analysis of DNA regulatory elements by synthetic saturation mutagenesis. Nat Biotechnol 27: 1173–1175 doi:10.1038/nbt.1589 19915551 10.1038/nbt.1589 PMC2849652

[48] Müller CA, Nieduszynski CA (2012) Conservation of replication timing reveals global and local regulation of replication origin activity. Genome Res doi:10.1101/gr.139477.112 10.1101/gr.139477.112 PMC3460190 22767388

[49] Müller CA, Hawkins M, Retkute R, Malla S, Wilson R, et al (2013) The dynamics of genome replication using deep sequencing. Nucleic Acids Res doi:10.1093/nar/gkt878 10.1093/nar/gkt878 PMC3874191 24089142

[50] Müller P, Park S, Shor E, Huebert DJ, Warren CL, et al (2010) The conserved bromo-adjacent homology domain of yeast Orc1 functions in the selection of DNA replication origins within chromatin. Genes Dev 24: 1418–1433 doi:10.1101/gad.1906410 20595233 10.1101/gad.1906410 PMC2895200

[51] Lantermann AB, Straub T, Strålfors A, Yuan G-C, Ekwall K, et al (2010) *Schizosaccharomyces pombe* genome-wide nucleosome mapping reveals positioning mechanisms distinct from those of *Saccharomyces cerevisiae* . Nat Struct Mol Biol 17: 251–257 doi:10.1038/nsmb.1741 20118936 10.1038/nsmb.1741

[52] Lubelsky Y, Sasaki T, Kuipers MA, Lucas I, Le Beau MM, et al (2011) Pre-replication complex proteins assemble at regions of low nucleosome occupancy within the Chinese hamster dihydrofolate reductase initiation zone. Nucleic Acids Res 39: 3141–3155 doi:10.1093/nar/gkq1276 21148149 10.1093/nar/gkq1276 PMC3082903

[53] Lee W, Tillo D, Bray N, Morse RH, Davis RW, et al (2007) A high-resolution atlas of nucleosome occupancy in yeast. Nat Genet 39: 1235–1244 doi:10.1038/ng2117 17873876 10.1038/ng2117

[54] Tsankov AM, Thompson DA, Socha A, Regev A, Rando OJ (2010) The role of nucleosome positioning in the evolution of gene regulation. PLoS Biol 8: e1000414 doi:10.1371/journal.pbio.1000414 20625544 10.1371/journal.pbio.1000414 PMC2897762

[55] Liang S, Wang B, Pan L, Ye Y, He M, et al (2012) Comprehensive structural annotation of *Pichia pastoris* transcriptome and the response to various carbon sources using deep paired-end RNA sequencing. BMC Genomics 13: 738 doi:10.1186/1471-2164-13-738 23276294 10.1186/1471-2164-13-738 PMC3547764

[56] Wang J, Zhuang J, Iyer S, Lin X, Whitfield TW, et al (2012) Sequence features and chromatin structure around the genomic regions bound by 119 human transcription factors. Genome Res 22: 1798–1812 doi:10.1101/gr.139105.112 22955990 10.1101/gr.139105.112 PMC3431495

[57] Yuan G-C, Liu Y-J, Dion MF, Slack MD, Wu LF, et al (2005) Genome-scale identification of nucleosome positions in *S. cerevisiae* . Science 309: 626–630 doi:10.1126/science.1112178 15961632 10.1126/science.1112178

[58] Hahn J-S, Hu Z, Thiele DJ, Iyer VR (2004) Genome-wide analysis of the biology of stress responses through heat shock transcription factor. Mol Cell Biol 24: 5249–5256 doi:10.1128/MCB.24.12.5249-5256.2004 15169889 10.1128/MCB.24.12.5249-5256.2004 PMC419887

[59] Trinklein ND, Murray JI, Hartman SJ, Botstein D, Myers RM (2004) The role of heat shock transcription factor 1 in the genome-wide regulation of the mammalian heat shock response. Mol Biol Cell 15: 1254–1261 doi:10.1091/mbc.E03-10-0738 14668476 10.1091/mbc.E03-10-0738 PMC363119

[60] Shirahige K, Iwasaki T, Rashid MB, Ogasawara N, Yoshikawa H (1993) Location and characterization of autonomously replicating sequences from chromosome VI of *Saccharomyces cerevisiae* . Mol Cell Biol 13: 5043–5056. 8336734 10.1128/mcb.13.8.5043-5056.1993 PMC360155

[61] Dujon B (2010) Yeast evolutionary genomics. Nat Rev Genet 11: 512–524 doi:10.1038/nrg2811 20559329 10.1038/nrg2811

[62] Harbison CT, Gordon DB, Lee TI, Rinaldi NJ, Macisaac KD, et al (2004) Transcriptional regulatory code of a eukaryotic genome. Nature 431: 99–104 doi:10.1038/nature02800 15343339 10.1038/nature02800 PMC3006441

[63] Gasser B, Maurer M, Rautio J, Sauer M, Bhattacharyya A, et al (2007) Monitoring of transcriptional regulation in *Pichia pastoris* under protein production conditions. BMC Genomics 8: 179 doi:10.1186/1471-2164-8-179 17578563 10.1186/1471-2164-8-179 PMC1919374

[64] Liachko I, Dunham MJ (2013) An autonomously replicating sequence for use in a wide range of budding yeasts. FEMS Yeast Res doi:10.1111/1567-1364.12123 10.1111/1567-1364.12123 PMC3959236 24205893

[65] Lee CC, Williams TG, Wong DWS, Robertson GH (2005) An episomal expression vector for screening mutant gene libraries in *Pichia pastoris* . Plasmid 54: 80–85 doi:10.1016/j.plasmid.2004.12.001 15907541 10.1016/j.plasmid.2004.12.001

[66] Fowler DM, Araya CL, Gerard W, Fields S (2011) Enrich: Software for Analysis of Protein Function by Enrichment and Depletion of Variants. Bioinformatics doi:10.1093/bioinformatics/btr577 10.1093/bioinformatics/btr577 PMC3232369 22006916

[67] Crooks GE, Hon G, Chandonia J-M, Brenner SE (2004) WebLogo: a sequence logo generator. Genome Res 14: 1188–1190 doi:10.1101/gr.849004 15173120 10.1101/gr.849004 PMC419797

[68] Bailey TL, Gribskov M (1998) Combining evidence using p-values: application to sequence homology searches. Bioinformatics 14: 48–54. 9520501 10.1093/bioinformatics/14.1.48

[69] Grant CE, Bailey TL, Noble WS (2011) FIMO: scanning for occurrences of a given motif. Bioinformatics 27: 1017–1018 doi:10.1093/bioinformatics/btr064 21330290 10.1093/bioinformatics/btr064 PMC3065696

[70] Huberman JA (1997) Mapping replication origins, pause sites, and termini by neutral/alkaline two-dimensional gel electrophoresis. Methods 13: 247–257 doi:10.1006/meth.1997.0524 9441851 10.1006/meth.1997.0524

[71] Adey A, Morrison HG, Asan, Xun X, Kitzman JO, et al (2010) Rapid, low-input, low-bias construction of shotgun fragment libraries by high-density in vitro transposition. Genome Biol 11: R119 doi:10.1186/gb-2010-11-12-r119 21143862 10.1186/gb-2010-11-12-r119 PMC3046479

