# Supplementary figures and images for: GC-Rich DNA Elements Enable Replication Origin Activity in the Methylotrophic Yeast Pichia pastoris
Source: PLoS Genet. 2014 Mar 6;10(3):e1004169. doi: 10.1371/journal.pgen.1004169 (PMC3945215; doi:10.1371/journal.pgen.1004169)

Figure S1

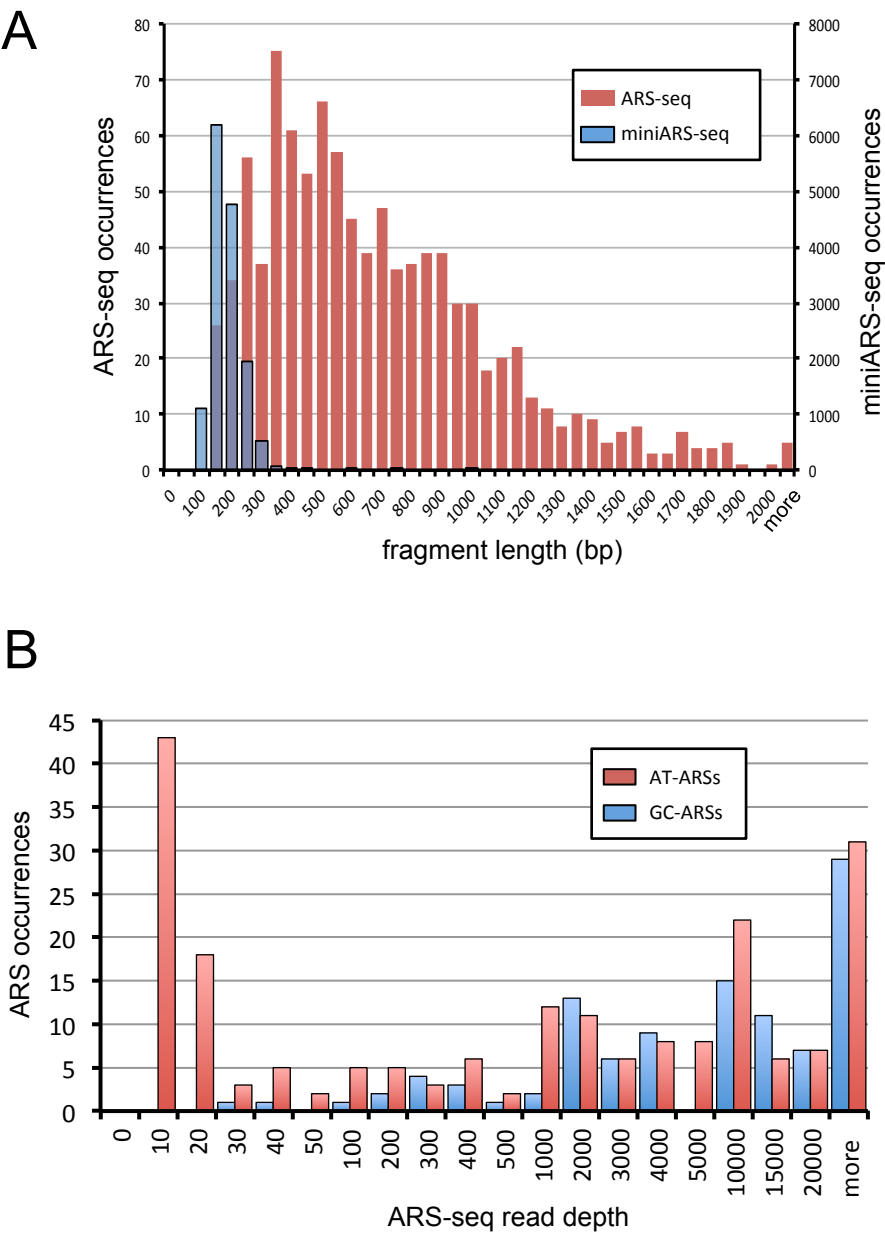

Supplement: Figure S1 — Summary of ARS-seq and miniARS-seq results. (A) ARS fragment length distributions. The distribution of lengths of unique ARS-seq and miniARS-seq inserts as shown in Tables S1 and S2. (B) ARS-seq read depth distributions. The combined read depths for all ARSs identified by ARS-seq are shown in red (AT-ARSs) and blue (GC-ARSs). The combined read depths for each ARS are available in Table S3. (PDF) [file pgen.1004169.s001.pdf]

Figure S2

| flanking genes | 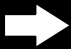 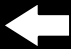 | 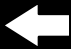 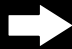 | 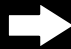 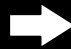 |
|----------------|---------------------------------------------------------------------------------------------------------------------------------------------------------------------|----------------------------------------------------------------------------------------------------------------------------------------------------------------------|-------------------------------------------------------------------------------------------------------------------------------------------------------------------------|
| All intergenes | 1378                                                                                                                                                                | 1463                                                                                                                                                                 | 2106                                                                                                                                                                    |
| GC-ARs         | 19 <sub>(30)</sub>                                                                                                                                                  | 29 <sub>(31)</sub>                                                                                                                                                   | 58 <sub>(45)</sub>                                                                                                                                                      |
| AT-ARs         | 27 <sub>(45)</sub>                                                                                                                                                  | 45 <sub>(47)</sub>                                                                                                                                                   | 89 <sub>(71)</sub>                                                                                                                                                      |

Supplement: Figure S2 — The direction of genes flanking intergenic ARSs. Intergenic regions >1 bp in length [31] are grouped based on the direction of transcription of the pair of genes flanking the intergenic space. Numbers of intergenes falling into convergent (left), divergent (middle), and tandem (right) orientations are shown (All intergenes). The numbers of GC-ARS or AT-ARS containing intergenes in different orientation groups are shown (GC-ARSs and AT-ARSs respectively). Numbers in parentheses refer to the number of intergenes expected in the given group assuming a random distribution. (PDF) [file pgen.1004169.s002.pdf]

Figure S3

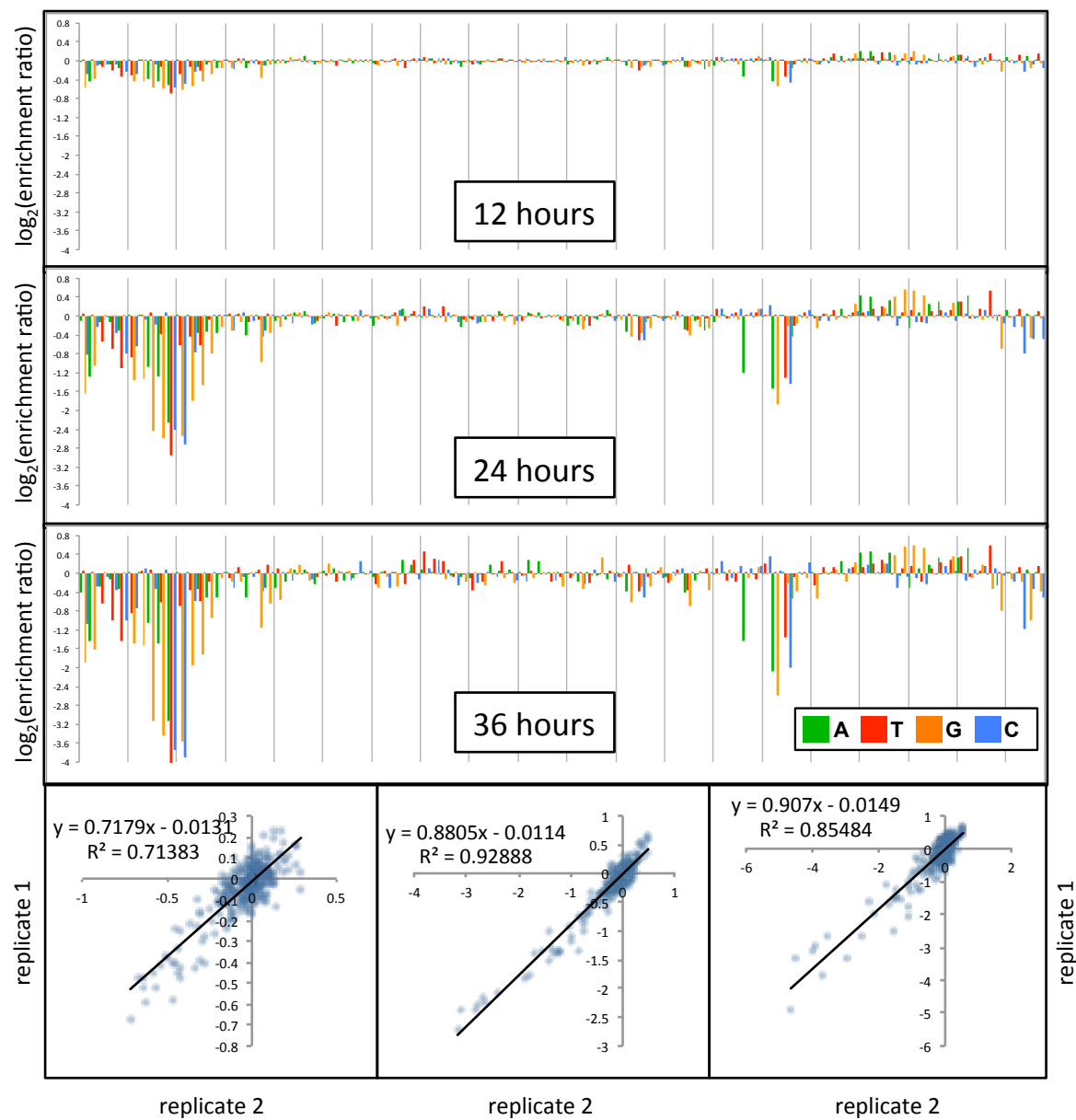

Supplement: Figure S3 — ARS-C379 mutARS-seq data during competitive growth. Data processed as described (Methods) is shown as the average of two replicates for 12-, 24-, and 36-hour timepoints normalized against the same input sample. Data are plotted on the same y-axis scale to aid visual comparison. Scatterplots show correlations between replicates of the same timepoint samples (lower panels). (PDF) [file pgen.1004169.s003.pdf]

Figure S4

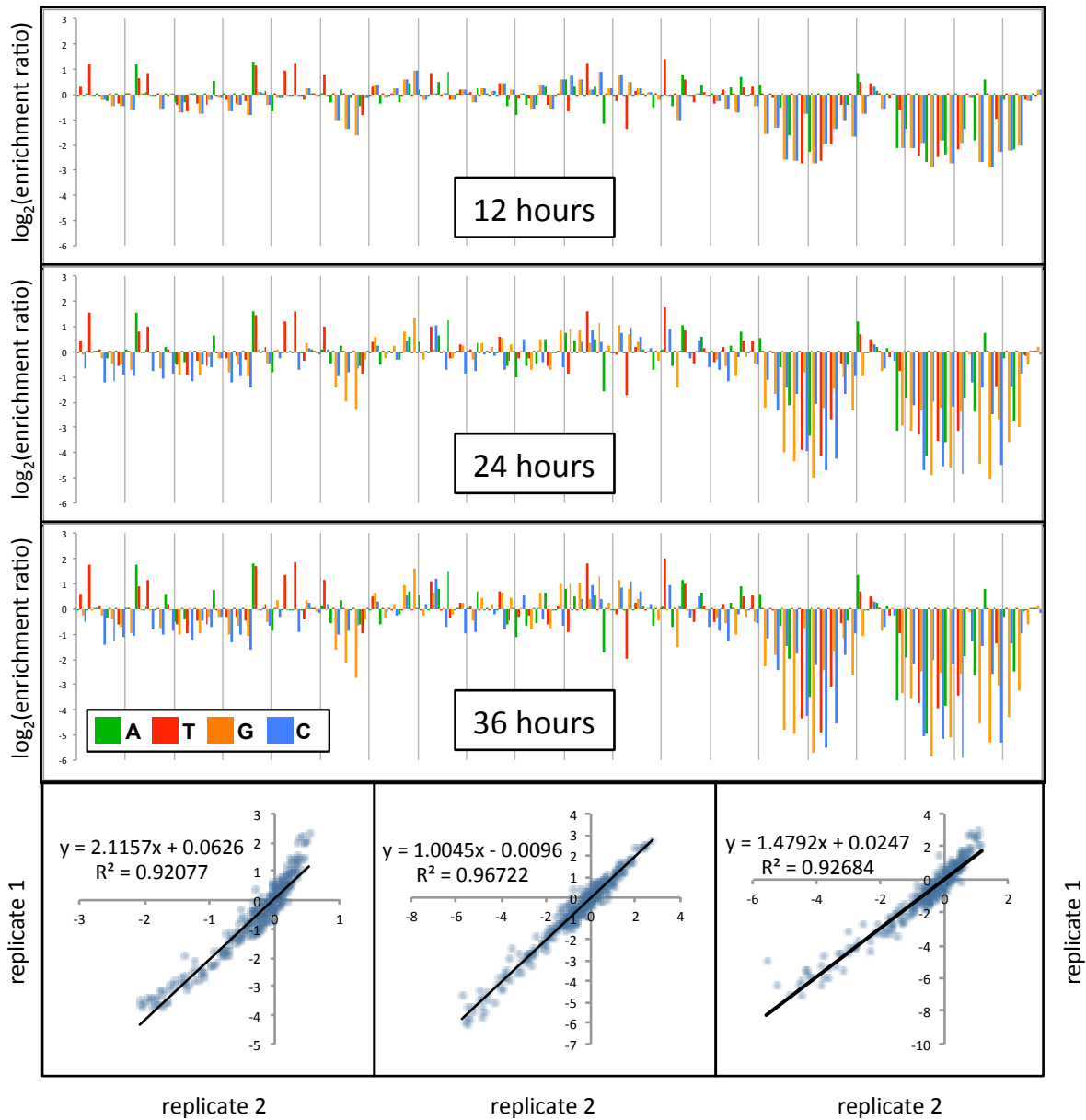

Supplement: Figure S4 — ARS-A2772 mutARS-seq data during competitive growth. Data processed as described (Methods) is shown as the average of two replicates for 12-, 24-, and 36-hour timepoints normalized against the same input sample. Data are plotted on the same y-axis scale to aid visual comparison. Scatterplots show correlations between replicates of the same timepoint samples (lower panels). (PDF) [file pgen.1004169.s004.pdf]

Figure S5

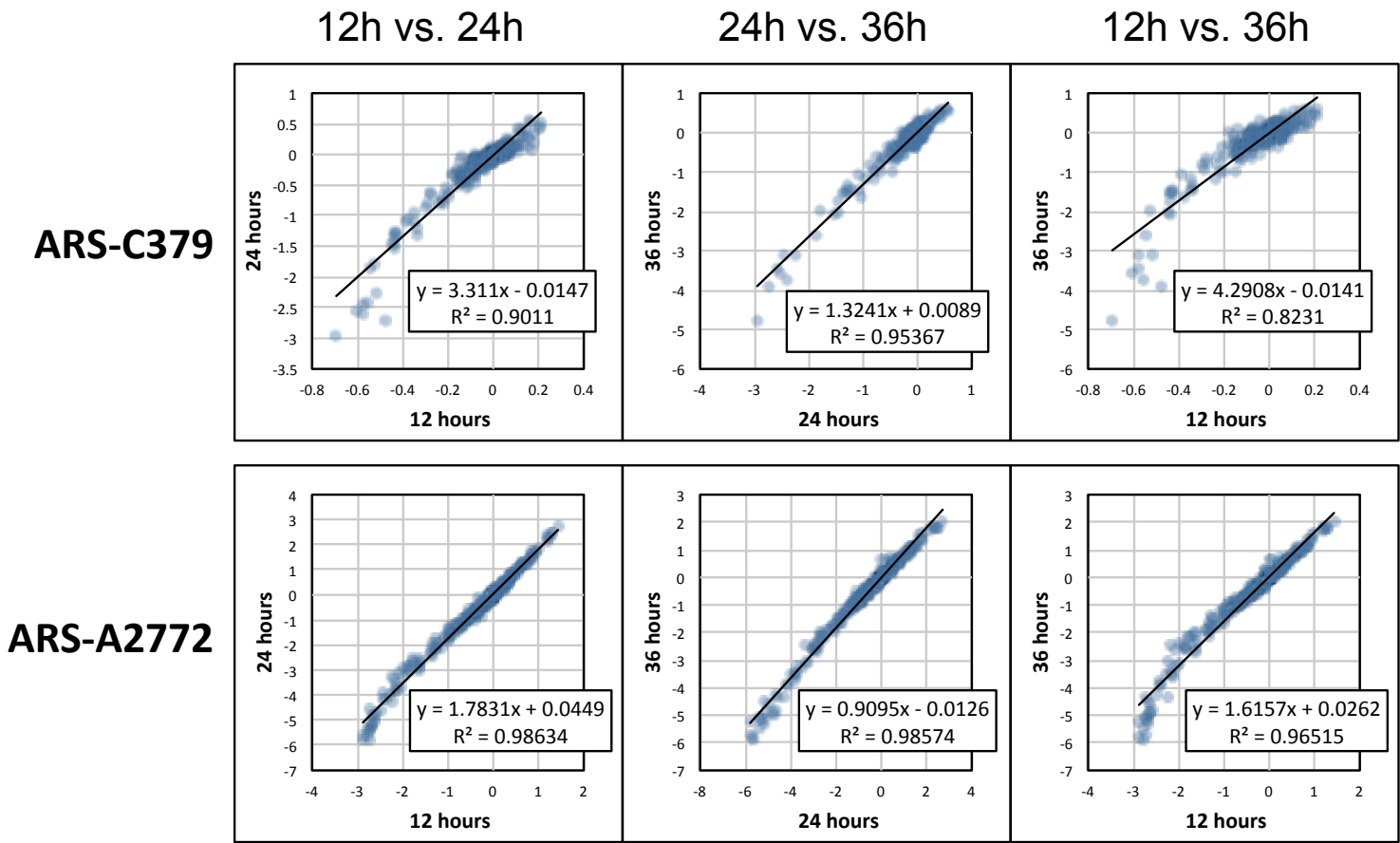

Supplement: Figure S5 — Comparisons of mutARS-seq data during competitive growth. Averaged mutARS-seq data from 12-, 24-, and 36-hour timepoints are plotted as scatterplots. (PDF) [file pgen.1004169.s005.pdf]

Figure S6

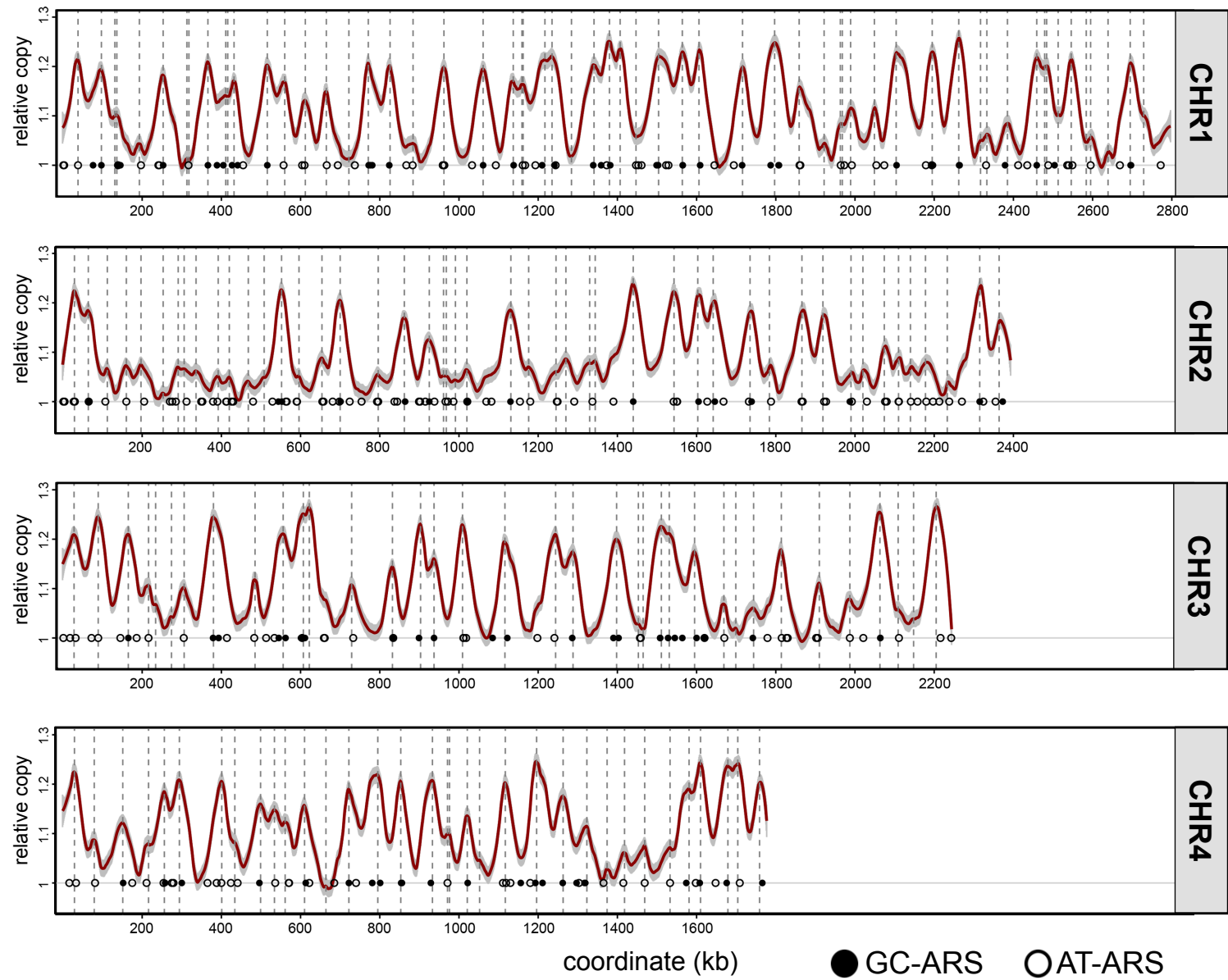

Supplement: Figure S6 — Replication profiles of all P. pastoris chromosomes. Replication timing profiles were computed as discussed (Figure 4) and are shown for all four P. pastoris chromosomes. Un-smoothed ratio data for one of the replicates is shown in grey. Locations of GC-ARSs and AT-ARSs are indicated by open and shaded circles respectively. (PDF) [file pgen.1004169.s006.pdf]

Figure S7

A

*P. pastoris*  
GC-ACS  
(forced 50bp)

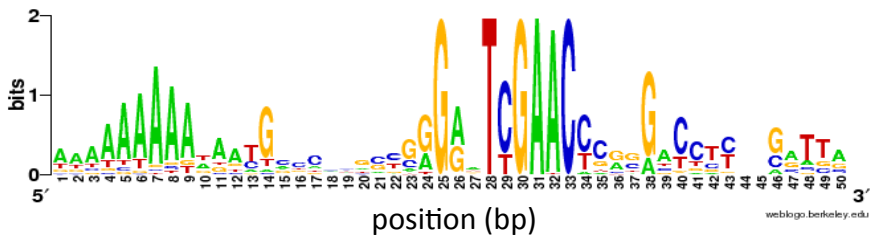

B

*P. pastoris* motif derived from  
mutARS-seq

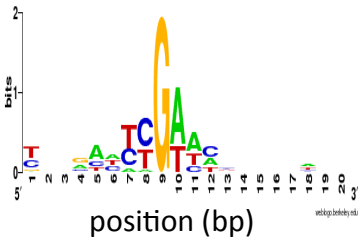

Supplement: Figure S7 — Comparison of GC-ARS motifs. (A) The ACS motif identified from 107 GC-ARSs when the motif length is forced to be 50 bp. (B) The motif obtained from mutARS-seq of ARS-C379 using a procedure identical to the one used to obtain the AT-rich motif in Figure 3C. (PDF) [file pgen.1004169.s007.pdf]

Figure S8

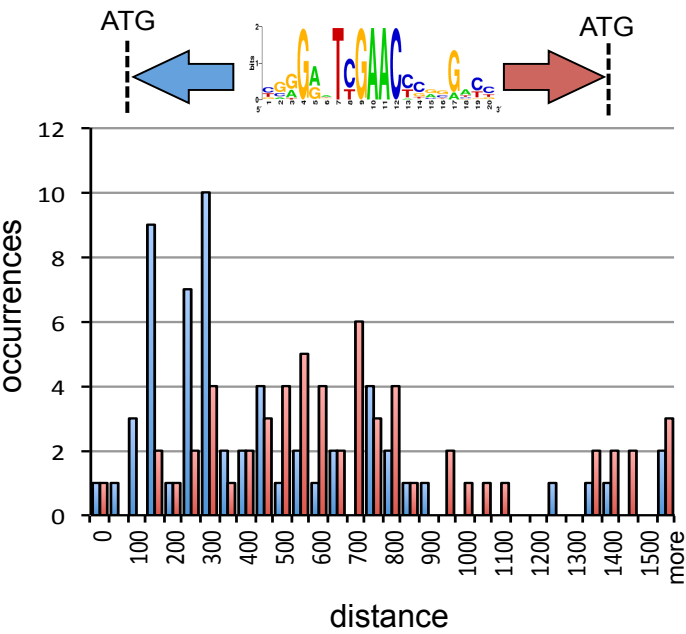

Supplement: Figure S8 — Distance between GC-ACS and flanking ATGs. For all instances where the GC-ARS is found adjacent to the 5′ end of a gene, we calculated the distance between the start ATG codon of the ORF and the closest edge of the GC-ACS match. The distributions of ATG-to-ACS distances are plotted as histograms based on the direction relative to the ACS. (PDF) [file pgen.1004169.s008.pdf]
